# Differences in structural MRI and diffusion tensor imaging underlie visuomotor performance declines in older adults with an increased risk for Alzheimer’s disease

**DOI:** 10.3389/fnagi.2022.1054516

**Published:** 2023-01-12

**Authors:** Alica Rogojin, Diana J. Gorbet, Kara M. Hawkins, Lauren E. Sergio

**Affiliations:** ^1^School of Kinesiology and Health Science, York University, Toronto, ON, Canada; ^2^Centre for Vision Research, York University, Toronto, ON, Canada; ^3^Vision: Science to Applications (VISTA) Program, York University, Toronto, ON, Canada

**Keywords:** visuomotor integration, early detection, Alzheimer’s disease, apolipoprotein e4, diffusion tensor imaging, grey matter

## Abstract

**Introduction:**

Visuomotor impairments have been demonstrated in preclinical AD in individuals with a positive family history of dementia and APOE e4 carriers. Previous behavioral findings have also reported sex-differences in performance of visuomotor tasks involving a visual feedback reversal. The current study investigated the relationship between grey and white matter changes and non-standard visuomotor performance, as well as the effects of APOE status, family history of dementia, and sex on these brain-behavior relationships.

**Methods:**

Older adults (*n* = 49) with no cognitive impairments completed non-standard visuomotor tasks involving a visual feedback reversal, plane-change, or combination of the two. Participants with a family history of dementia or who were APOE e4 carriers were considered at an increased risk for AD. T1-weighted anatomical scans were used to quantify grey matter volume and thickness, and diffusion tensor imaging measures were used to quantify white matter integrity.

**Results:**

In APOE e4 carriers, grey and white matter structural measures were associated with visuomotor performance. Regression analyses showed that visuomotor deficits were predicted by lower grey matter thickness and volume in areas of the medial temporal lobe previously implicated in visuomotor control (entorhinal and parahippocampal cortices). This finding was replicated in the diffusion data, where regression analyses revealed that lower white matter integrity (lower FA, higher MD, higher RD, higher AxD) was a significant predictor of worse visuomotor performance in the forceps minor, forceps major, cingulum, inferior fronto-occipital fasciculus (IFOF), inferior longitudinal fasciculus (ILF), superior longitudinal fasciculus (SLF), and uncinate fasciculus (UF). Some of these tracts overlap with those important for visuomotor integration, namely the forceps minor, forceps major, SLF, IFOF, and ILF.

**Conclusion:**

These findings suggest that measuring the dysfunction of brain networks underlying visuomotor control in early-stage AD may provide a novel behavioral target for dementia risk detection that is easily accessible, non-invasive, and cost-effective. The results also provide insight into the structural differences in inferior parietal lobule that may underlie previously reported sex-differences in performance of the visual feedback reversal task.

## 1. Introduction

Alzheimer’s disease (AD) is a slowly progressive neurodegenerative disease and the most common cause of dementia in older adults. AD pathology begins several decades before the onset of clinical dementia, and symptoms appear only after there has already been significant damage to the brain (Morris, 2005), which means the assessment of increased dementia risk in the early stages of AD is crucial. Recently published guidelines suggest that effective interventions could be initiated during this early stage of disease progression (Dubois et al., 2016). Even in the absence of effective disease-modifying treatments that prevent neuronal loss and resultant cognitive decline (Vaz and Silvestre, 2020), the early recognition of dementia risk is important for improving patient outcomes now and in the future when effective therapies become available. Current clinical diagnosis of AD involves procedures that can be invasive, expensive, and time-ineffective (such as cerebrospinal fluid analysis, blood tests, and neuroimaging), making their use as frontline screening tools limited. Furthermore, studies have shown that dementia is under-diagnosed in primary care (Connolly et al., 2011; Lang et al., 2017; Amjad et al., 2018) even as the prevalence of AD is projected to rise due to increased life expectancy (Alzheimer’s Association, 2019). A number of medical, environmental, and lifestyle factors have been associated with an increased risk for developing mild cognitive impairment and dementia, some of which include modifiable risk factors such as diabetes, tobacco use, physical inactivity, obesity, and social isolation (Livingston et al., 2020; Litke et al., 2021). These factors highlight the need for the development of a non-invasive and cost-effective assessment tool for those at increased dementia risk. By identifying individuals at a greater risk for developing dementia, healthcare providers can provide evidence-based recommendations on lifestyle changes, interventions, and management of physical and mental health conditions to delay or slow down the progression of AD (World Health Organization, 2019).

As an alternative to current diagnostic tools and procedures, measuring the dysfunction of brain networks underlying visuomotor transformations in early-stage AD may provide a novel behavioral target for dementia risk detection that is easily accessible and cost-effective. Default or “standard” visually-guided arm movements typically involve looking towards the intended target of a reach, followed by an arm movement to the same location (Gielen et al., 1984; Wise et al., 1996; Helsen et al., 1998). For example, reaching directly towards a cup of tea to pick it up. However, many tasks in our everyday lives require us to spatially dissociate the targets of the eye and arm movements, such as during a plane-change or a visual feedback reversal. A common example of a plane-change is using a computer with a mouse or trackpad. Arm movements controlling the mouse or trackpad are made along the horizontal plane in order to move a cursor on the vertical computer screen. An example of a visual feedback reversal is seen when using some computer trackpads, where sliding up on the trackpad scrolls the page down. Another example would be the use of a car’s rear-view camera (or, rear-view mirror) while backing up. To avoid hitting any objects, one must turn the steering wheel leftward in a vertical plane while the object on the screen (or, in the mirror) moves to the right. Here, the plane of limb movement is decoupled from the guiding visual information, and there is a visual feedback reversal as well. These decoupled or “non-standard” visually-guided arm movements require the integration of some form of cognitive information into the visuomotor transformation, and the mapping between the visual stimulus and response must be learned and calibrated (Wise et al., 1996). Some patient populations show an impaired ability to successfully perform non-standard visuomotor tasks, even while their performance on standard mapping tasks remains unaffected. These impairments have been observed in patients with mild cognitive impairment (Salek et al., 2011), in the early stages of dementia due to AD (Tippett et al., 2012; de Boer et al., 2014, 2015, 2016), and in individuals at an increased risk for future dementia (due to a dementia family history, or a genetic risk due to being an APOE e4 carrier) who did not yet show any cognitive deficits (Hawkins and Sergio, 2014; Lu et al., 2021).

Visuomotor integration relies on interconnected brain regions within the frontoparietal network (Wise et al., 1997; Sabes, 2000; Filimon, 2010; Caminiti et al., 2017). There are some areas of activity common to both standard and non-standard visuomotor reaching tasks including the contralateral primary, premotor, and medial motor regions, as well as the postcentral gyrus (Gorbet et al., 2004). Additional brain regions become active during non-standard reaching tasks as visuomotor transformations become increasingly dissociated. These brain areas include the anterior prefrontal cortex, precuneus, and large regions of the occipital lobe for non-standard visuomotor integration involving a plane-change (Gorbet and Sergio, 2018), as well as areas of the premotor, primary motor and somatosensory, posterior parietal, middle occipital, and medial occipital cortices during a visual feedback reversal task (Gorbet and Sergio, 2016). When contrasting a standard task with a non-standard task involving both a feedback reversal and plane-change using a joystick, the non-standard task showed increased activity in the left precuneus, the right superior frontal and middle temporal gyri, and bilaterally in the angular gyri (Gorbet et al., 2004). The precuneus and inferior parietal lobule in particular appear to be important for discriminating between standard and non-standard task conditions (Gorbet and Sergio, 2016, 2018).

Given the known functional substrates of non-standard visuomotor transformations, several white matter tracts that connect these areas of the frontoparietal network may be implicated in visually-guided reaching. The superior longitudinal fasciculus (SLF) has been shown to be important for visuomotor integration (Rodríguez-Herreros et al., 2015; Budisavljevic et al., 2017) and to a lesser extent, the inferior fronto-occipital fasciculus (IFOF) which has been implicated in visuospatial attention and goal-directed behavior (de Schotten et al., 2011;Waller et al., 2017; Herbet et al., 2018). Both the SLF and IFOF have connections with the inferior parietal lobule (de Schotten et al., 2011; Burks et al., 2017), which is one of the regions important for non-standard visuomotor mapping. The inferior longitudinal fasciculus (ILF) runs between the anterior temporal and posterior occipital lobes (Bennett et al., 2012; Wycoco et al., 2013), and is involved in processing visual cues and thus also in visually-guided decisions and goal-oriented behaviors (Waller et al., 2017; Herbet et al., 2018). A meta-analysis reported that the corpus callosum also plays a central role in visuomotor integration, as well as in higher-order cognitive functions such as spatial attention and executive control (Schulte and Müller-Oehring, 2010).

Little evidence exists regarding the role of the medial temporal lobe in visual guidance of movements and in visuomotor or motor tasks. Some areas of the medial temporal lobe have been implicated in visuomotor coordination. The parahippocampal cortex may be an important relay for the transformation between visual inputs and hand kinematics (outputs), and the hippocampus, parahippocampus, and entorhinal cortex appear to encode the position of the hand in space (Tankus and Fried, 2012). The retrosplenial cortex has extensive connections with the parietal cortex (Vann et al., 2009), playing a central role in the coordination of information between internal and external spatial frames of reference (Clark et al., 2018) which is important for visually-guided movements. Although dramatic neuronal loss is not observed in preclinical AD, there is mild grey matter atrophy in areas of the medial temporal lobe including the entorhinal, perirhinal, hippocampal, and parahippocampal cortical regions during this stage (Honea et al., 2009, 2011). There is increasing evidence that hippocampal subregions are differentially affected by AD progression, with the earliest atrophy found in the subiculum and CA1 subfields in preclinical AD (Apostolova et al., 2010; Donix et al., 2010a,b). The CA1 has reciprocal connections with the inferior parietal lobule (Rockland and Van Hoesen, 1999; Clower et al., 2001). The retrosplenial cortex is also affected early in AD progression (Nestor et al., 2003).

Diffusion tensor imaging measures (fractional anisotropy, FA; mean diffusivity, MD; radial diffusivity, RD; and axial diffusivity, AxD) provide information about white matter microstructure and pathology (Song et al., 2002, 2003), and have been used as an index of white matter integrity (Pfefferbaum et al., 2000; Acosta-Cabronero and Nestor, 2014; Molloy et al., 2021). Several studies have suggested that white matter damage may precede grey matter atrophy in AD (Hong et al., 2016; Hoy et al., 2017; Dadar et al., 2020). White matter integrity declines have been found in several major white matter tracts including the corpus callosum, cingulum, SLF, IFOF, ILF, and uncinate fasciculus (UF) in cognitively healthy adults who are APOE e4 carriers and have a parental family history of AD (Bendlin et al., 2010; Gold et al., 2012; Westlye et al., 2012; Adluru et al., 2014). Some of these tracts overlap with those mentioned previously to be involved in visuomotor integration. Known early AD pathology (Thal et al., 2002; Mintun et al., 2006; Pletnikova et al., 2018) and atrophy (Chelat et al., 2010; Jacobs et al., 2011; Doré et al., 2013) in frontal and parietal regions, combined with frontoparietal tract involvement in healthy rule-based visuomotor integration, suggest that reduced communication along these tracts may underlie impaired performance in those at an increased risk for dementia.

In the present study, we first examined whether individuals at an increased risk for AD (due to a family history or a genetic risk) showed grey matter atrophy or declines in white matter integrity in brain regions and tracts typically affected early in disease progression. However, the main objective was to explore the neural underpinnings to previous behavioral findings, where individuals with a positive family history of dementia and APOE e4 carriers demonstrated performance deficits on non-standard visuomotor tasks (Rogojin et al., 2019). There were also sex-related differences in performance of the non-standard visuomotor task involving a feedback reversal. In the current study, we examined the neural anatomy underlying impaired non-standard visuomotor performance, and whether this impairment is affected by family history of AD, APOE e4 status, or sex. We hypothesized a positive relationship between brain structural and white matter integrity measures and visuomotor performance. We predicted grey matter atrophy within the inferior parietal lobule, precuneus, and retrosplenial cortex to be associated with deficits in non-standard visuomotor integration. We were also interested in exploring whether atrophy in the areas of the medial temporal lobe that have been previously implicated in visuomotor coordination would be predictive of worse visuomotor task performance. Based on the importance of the SLF, IFOF, ILF, and corpus callosum in visuomotor integration and goal-directed behavior, we also predicted that white matter integrity declines in these tracts would similarly be associated with declines in non-standard visuomotor integration performance. An exploratory component to the study was to explore the effects of family history, APOE status, and sex on these brain-behavior relationships.

## 2. Methods

### 2.1. Participants

Forty-nine right-handed older adults aged 49 to 69 were included in the current study: 25 individuals with a positive family history of dementia but with no cognitive impairment (*n* = 12 females), and 24 individuals with no family history of dementia (*n* = 12 females; see Table 1 for demographic information). Classification of a positive family history of dementia was based on self-reported maternal or multiple family history (including at least one first-degree relative) of late-onset AD or probable AD. Cognitive function was measured with the Montreal Cognitive Assessment (MoCA), where scoring at or above education-adjusted norms of 26 or higher indicated no cognitive impairment. A higher risk for AD is associated with a maternal history of dementia, while paternal history does not confer the same increased risk and was therefore not used for high-dementia risk classification (Honea et al., 2010, 2011). Participants were excluded if their parents were deceased at a young age before dementia could be detected, or if the participant was estranged from either of their biological parents and did not know their medical history. Classification of no family history of dementia was based on participants having no family history of AD or any other type of dementia, not demonstrating memory impairments outside of their age range norm, and scoring at or above age-average on the MoCA. Participants with a positive family history of dementia were age-balanced with participants without a family history of dementia. At present, the apolipoprotein E (APOE) e4 allele represents the strongest and best-established genetic risk factor for progression to clinical AD (Reitz and Mayeux, 2014; Dubois et al., 2016). Participants with a family history of dementia or who were APOE e4 carriers were considered at an increased risk for AD. The exclusion criteria included uncorrected visual impairments, upper-limb impairments, any medical conditions that would hinder motor task performance (e.g., severe arthritis or dystonia), neurological illnesses (e.g., Parkinson’s disease, depression, schizophrenia, alcoholism, epilepsy), history of head injury (e.g., mild, severe), stroke, and medical diagnoses that might impact white matter integrity and brain connectivity (i.e., hypertension or diabetes). The study protocol was approved by the Human Participants Review Sub-Committee of York University’s Ethics Review Board.

**Table 1:** Participant demographic features.

	APOE e4 positive	APOE e4 negative	FH+	FH−	Females	Males
Demographics						
*n*	16	31	25	24	24	25
Age (years)	59.3 ± 5.30	58.4 ± 5.99	58.5 ± 6.10	58.8 ± 5.29	58.4 ± 5.81	58.8 ± 5.63
*Range*	*51–68*	*49–69*	*51–69*	*49–67*	*50–68*	*49–69*
FH+	11 (69%)	14 (45%)			12 (50%)	13 (52%)
APOE e4 positive			11 (69%)	5 (31%)	11 (46%)	5 (20%)
MoCA score	27.9 ± 1.5	28 ± 1.56	27.8 ± 1.52	28 ± 1.56	28.2 ± 1.53	27.6 ± 1.5
*Range*	*26–30*	*26–30*	*26–30*	*26–30*	*26–30*	*26–30*
Years of education	16.0 ± 3.70	17.7 ± 3.15	17.3 ± 3.52	16.8 ± 3.19	17.3 ± 3.07	16.7 ± 3.61
*Range*	*11–23*	*12–24*	*11–23*	*12–24*	*12–24*	*11–22*

FH+, positive family history of dementia; FH−, no family history of dementia; APOE, apolipoprotein E; MoCA, Montreal Cognitive Assessment.

### 2.2. APOE genotyping

A total of 2 ml of saliva were collected from each participant in microtubes from Diamed Lab Supplies Inc. using collection aids from Cedarlane Labs. Samples were sent to DNA Genotek Inc. (Ottawa, ON, Canada) for DNA extraction and APOE genotyping. DNA was isolated from samples according to the manufacturer’s protocols. Genotyping for APOE involved single nucleotide polymorphism (SNP) genotyping, and tested for SNPs rs429358 and rs7412. The proteins that are produced by the APOE gene are either E2, E3, or E4 combinations (for instance, E2/E3). The breakdown of the APOE genotyping in female participants was as follows: e3/e3 (*n* = 12), e3/e4 (*n* = 11). The breakdown of the APOE genotyping in male participants was as follows: e2/e3 (*n* = 1), e3/e3 (*n* = 18), e2/e4 (*n* = 1), e3/e4 (*n* = 4). One female and one male participant were excluded from statistical analyses looking at the APOE genotype as their genotyping results were inconclusive.

### 2.3. Behavioral data

#### 2.3.1. Behavioral data acquisition

**Figure 1:**
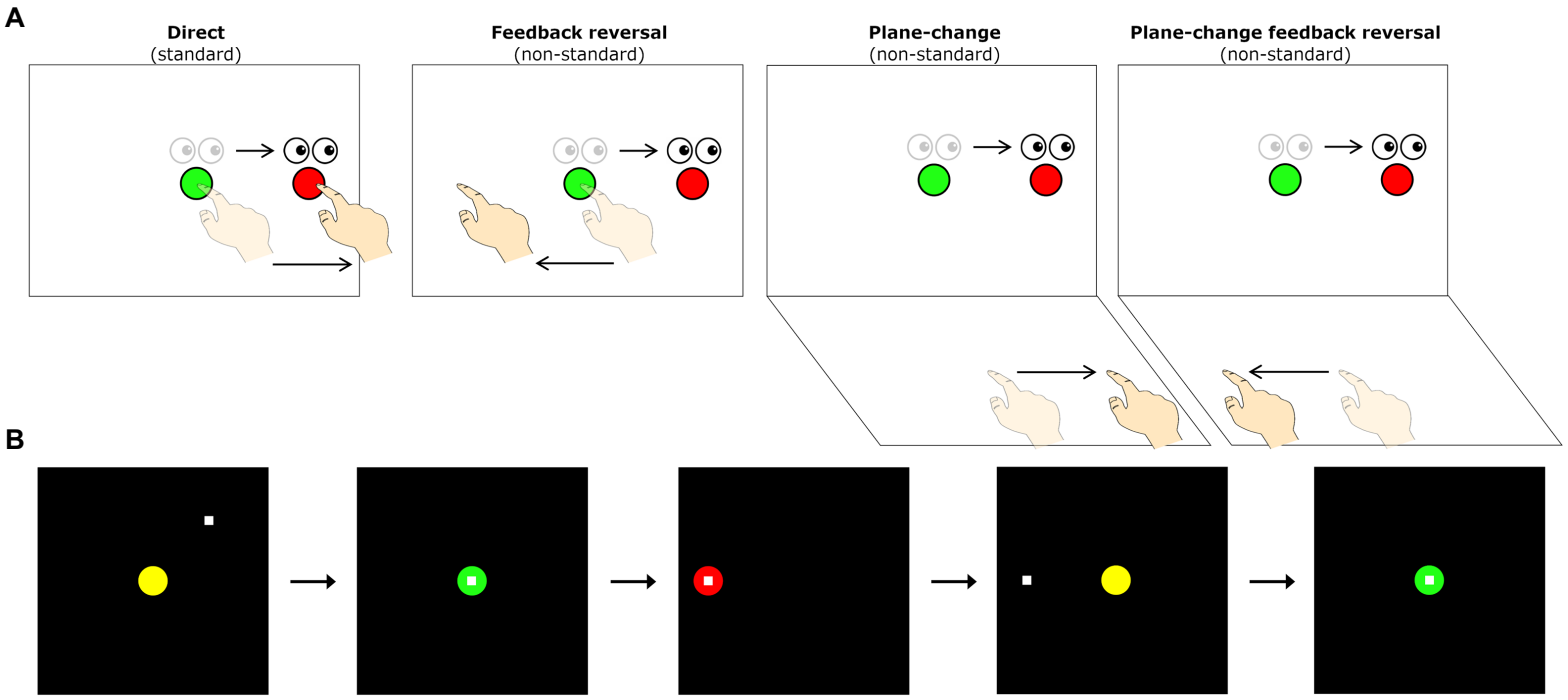
**(A)** Schematic drawing of the visuomotor task conditions. Lighter eye and hand symbols denote the starting position for each trial (green central target). Darker eye and hand symbols denote the instructed eye and hand movements for each task. Red circles denote the peripheral (reach) target, presented randomly in one of four locations (left, up, right, or down relative to the central target). The direct interaction task requires standard mapping, where participants slide their finger on a touch screen to move a cursor from a central target to one of four peripheral targets. The other three conditions involve non-standard visuomotor integration, where targets either have a 180° feedback reversal (feedback reversal), are spatially dissociated from the plane of hand motion (plane-change), or both (plane-change + feedback reversal). **(B)** Sequence of events during one trial of the visuomotor task. All trials begin in the central (home) target. Once the participant moves the cursor (denoted by the white square) into the central target, the target changes from yellow to green to signify a movement preparation period. After 4,000 ms, a red peripheral target appears in one of four directions (up, down, left, or right of the center) and serves as the ‘Go’ signal. Once the peripheral target is acquired and held for 500 ms it disappears, signaling the end of the trial. After an inter-trial interval of 2000 ms, the central yellow target reappears, and the participant moves back to the central target to initiate the next trial.

A detailed description of our visuomotor assessment has been previously published (Rogojin et al., 2019). Briefly, all subjects completed four visuomotor transformation tasks, similar to those previously used by our laboratory (Tippett and Sergio, 2006; Tippett et al., 2007; Salek et al., 2011; Tippett et al., 2012; Hawkins and Sergio, 2014, 2016; Hawkins et al., 2015). These tasks were found to discriminate between women at high- and low-AD risk with a classification accuracy of 86.4% (sensitivity: 81.8%, specificity: 90.9%; Hawkins and Sergio, 2014). The tasks involved making simple sliding finger movements between targets displayed on an Acer Iconia 6,120 dual-touchscreen tablet. These tasks were divided into one standard mapping condition (gaze and movement were coupled) and three different non-standard mapping conditions (gaze and movement were decoupled). In all four conditions, participants were instructed to slide the index finger of their right hand along the touch screen (either the vertical or horizontal screen depending on the condition) in order to displace the cursor from a central target to one of four peripheral targets (up, down, left, right) as quickly and as accurately as possible. In the standard mapping task (S), the spatial location of the visual target and the required hand movement were the same. The non-standard mapping tasks involved the finger movements being made either on a different plane (plane-change, PC), in the opposite direction (feedback reversal, FR), or both (PC + FR), from the spatial target location (see Figure 1A for depictions of all four visuomotor transformation task conditions). Eye movements were the same across all conditions (i.e., always to the guiding visual target on the vertical screen).

The four conditions were presented in randomized blocks, each consisting of five pseudo-randomly presented trials to each of the four peripheral targets. Peripheral targets were located 75 mm from the central target, with target diameters set to 20 mm. The tasks were displayed on a 170 × 170 mm black square and a surrounding grey background. There was a total of 20 trials per condition, and thus each participant completed a total of 80 trials across the four conditions. To ensure task comprehension, each participant was given two practice trials per peripheral target prior to each of the four conditions. The trial timings and participant movements consisted of the following steps: (1) a yellow central (home) target was presented on the vertical tablet, (2) participants moved a white cursor to the central target, changing its color to green once they reached it, (3) after holding the central target for 4,000 ms, one of four red peripheral targets appeared and the central target disappeared, serving as the ‘Go’ signal for initiation of a movement, (4) participants were told to look towards the visual target and slide their finger along the touchscreen to direct the cursor towards the target, (5) once the peripheral target was reached and the participant held it for 500 ms, it disappeared, signaling the end of the trial, (6) the next trial began with the presentation of the central target after an inter-trial interval of 2000 ms (see Figure 1B for visual representations of a single trial completion).

In the standard condition, participants were asked to slide their finger directly to the target on the vertical screen (the cursor was directly under their finger). In the PC condition (non-standard), participants needed to move on the horizontal screen while looking at the vertical screen in order to direct the cursor towards the visual target displayed on the vertical screen. In the FR condition (non-standard), the cursor moved in the opposite direction of the participant’s finger movements, requiring them to slide their finger on the vertical screen away from the visual target in order to move the cursor towards it. Finally, in the PC + FR condition (non-standard), movements needed to be made in the opposite direction and on a different plane from the visual target in order to direct the cursor towards it.

#### 2.3.2. Behavioral data preprocessing

Kinematic measures, including timing, finger position (x, y coordinates; 50 Hz sampling rate), and error data were recorded for each trial and converted into a MATLAB readable format using a custom written (C++) application. Custom analysis software (Matlab, Mathworks Inc.) was used to process individuals’ finger trajectories with a fourth-order (dual pass) low-pass Butterworth filter at 10 Hz. Finger trajectories were generated from these filtered paths for each successful trial and displayed on a Cartesian plot illustrating finger location data superimposed on central and peripheral target locations. Movement onsets and ballistic movement offsets (the initial movement prior to any corrective movements) were scored at 10% peak velocity. Total movement offsets were scored as the final 10% peak velocity point once the finger position was within the correct peripheral target. If the initial movement successfully resulted in the finger reaching the peripheral target, then ballistic and total movement offsets were the same. These movement profiles were then verified by visual inspection, and manually corrected when necessary. Unsuccessful trials (error data) were detected by the data collection software by meeting the following criteria: finger left the home target too early (<4,000 ms), reaction time (RT) was <150 or >8,000 ms, or total movement time was >10,000 ms. Trials in which the first ballistic movement exited the boundaries of the central target in the wrong direction (>90^o^ in either direction from a straight line to the target) were coded as direction reversals. Direction reversals were not included in metrics from correct trials, but instead were analyzed as a separate variable. All scored data were then processed to compute 7 different timing, accuracy, and precision measures described below. Any trials exceeding 2 standard deviations from the participant’s mean for any of the outcome measures were eliminated from final outcome calculations.

The kinematic outcome measures were as follows: (1) Reaction time (RT), the time interval (in ms) between the central target disappearance and movement onset; (2) Full movement time (MTf), the time (in ms) between movement onset and offset; (3) Peak velocity (PV), the maximum velocity obtained during the ballistic movement, and used to calculate the 10% threshold used for determining movement onsets and offsets; (4) Path length (PL), the total distance (in mm, calculated from the x and y trajectories) travelled between movement onset and offset; (5) Absolute error (AE), a measure of end-point accuracy, and the average distance (in mm) from the individual ballistic movement endpoints (∑ x/n, ∑ y/n) to the actual target location; (6) Variable error (VE), a measure of end-point precision, and the distance (in mm) between the individual ballistic movement endpoints (σ) from their mean movement; and (7) Direction reversals (DR), recorded as a percentage of total completed trials. Corrective path length (CPL) represents corrective movements and was quantified by subtracting the PLb (initial movement offset) from the PLf (full movement offset).

### 2.4. Magnetic resonance imaging

#### 2.4.1. Image acquisition

MRI data were acquired using a 3 Tesla (3 T) Siemens Trio scanner at York University. Participants received a T1-weighted anatomical scan using a sagittal volumetric magnetization-prepared rapid gradient echo (MP-RAGE) sequence. The MP-RAGE consisted of the following acquisition parameters: 192 sagittal slices (slice thickness of 1 mm, with no gap), field of view (FOV) of 256 × 256 mm, matrix size of 256 × 256 resulting in a voxel resolution of 1 × 1 × 1 mm, echo time (TE) = 2.96 ms, repetition time (TR) = 2,300 ms, flip angle = 9^o^. The MP-RAGE scan was used to quantify grey matter volume and thickness. For assessing white matter integrity, a whole-brain diffusion-weighted scan was acquired with 64 encoding directions using diffusion-weighted spin-echo single-shot echo planar imaging. The diffusion tensor imaging (DTI) sequence used the following acquisition parameters: 56 axial slices (slice thickness of 2 mm, with no gap), FOV of 192 × 192 mm, matrix size of 128 × 128 resulting in a voxel resolution of 1.5 × 1.5 × 2.0 mm, TE = 86 ms, TR = 6,900 ms, b-value = 1,000 s/mm^2^ (including one volume with no diffusion gradient, b = 0 s/mm^2^).

#### 2.4.2. Structural MRI

**Preprocessing:** The cortical surface was reconstructed using FreeSurfer 7.1 (Harvard Medical School, Boston, United States; https://surfer.nmr.mgh.harvard.edu/) with individual T1-weighted MR images serving as input. The main Freesurfer reconstruction pipeline (“*recon-all*”) was used to parcellate and segment the brain into anatomically distinct regions of the cortex and subcortical nuclei, respectively. Visual inspection of each participant’s parcellation and segmentation was performed to ensure that there were no obvious errors.

**Figure 2:**
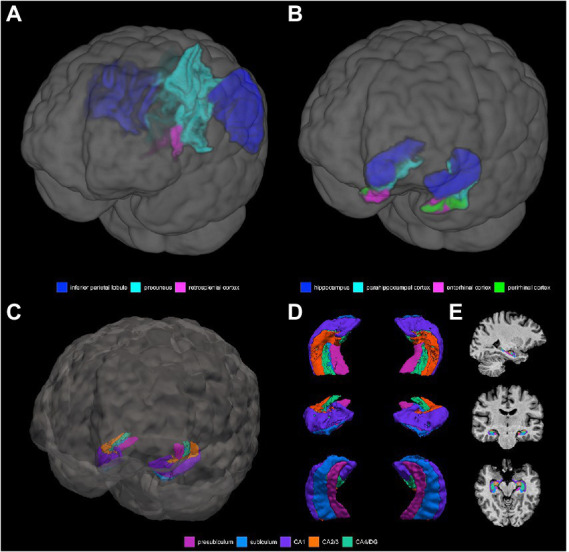
3D depictions of **(A)** areas important for non-standard visuomotor integration, and **(B)** areas of the medial temporal lobe, used for grey matter volume and cortical thickness analyses. Hippocampal subfields obtained from FreeSurfer segmentation and used for analysis are shown in **(C)** a 3D brain, **(D)** 3D superior, anterior, and inferior views, and **(E)** sagittal, coronal, and axial slices.

**Grey matter volume and thickness** (Figures 2A,B): Volume and thickness measures for the precuneus, inferior parietal lobule, and parahippocampal cortex were obtained from the Desikan-Killiany atlas (Desikan et al., 2006) used in *recon-all*. Volume and thickness measures for the entorhinal cortex and perirhinal cortex were obtained from the cytoarchitecturally-defined *ex vivo* labels in FreeSurfer. The retrosplenial cortex was defined as the posterior-ventral part of the cingulate gyrus, or the isthmus of the cingulate gyrus, from the Destrieux atlas (Destrieux et al., 2010) used in *recon-all*. The posterior-ventral part of the cingulate gyrus provides a mask slightly larger than Brodmann areas 29 and 30 (i.e., the retrosplenial cortex proper; Shine et al., 2016; Calati et al., 2018).

**Hippocampal subfield segmentation** (Figures 2C–E): Segmentation of the hippocampus into its subfields was performed with the FreeSurfer hippocampal module using the T1-weighted volumes processed with recon-all (Iglesias et al., 2015). This tool uses a probabilistic atlas constructed from *ex vivo* MRI data and a separate *in vivo* MRI dataset to automatically segment the hippocampal substructures. The resultant hippocampal subfields are the parasubiculum, presubiculum, subiculum, CA1, CA2/3, CA4/dentate gyrus (DG), molecular layer, fimbria, hippocampal fissure, hippocampus-amygdala-transition-area (HATA), and hippocampal tail. Visual inspection of each participants’ hippocampal subfield segmentation was performed to ensure that there were no obvious errors, with the caveat that precise visualization of the distinct subfield boundaries at the spatial resolution used in this study is difficult. The following subfields were excluded: the parasubiculum (volume of <100 μl and may be more prone to noise), the molecular later (at risk of partial volume effects), HATA (volume of <100 μl and may be more prone to noise), fimbria (thin white matter whose segmentation is less reliable and is at risk of partial volume effects), the hippocampal fissure (a sulcus containing a thin layer of cerebrospinal fluid, rather than a hippocampal substructure exactly, whose segmentation is less reliable), and the hippocampal tail (a region where the subfields are not histologically distinct and discernible from each other; Van Leemput et al., 2009; Kühn et al., 2012; Iglesias et al., 2015; Parker et al., 2019). The following 5 subfields were therefore used for the final analysis: the presubiculum, the subiculum, CA1, CA2/3, and CA4/DG.

**Intracranial volume adjustment**: Intracranial volume (ICV) adjustment is an important step as it takes individuals’ variations in head size into account (i.e., the confound that subjects with large ICV and/or of younger age would be expected to have a larger hippocampus). ICV was calculated from the T1-weighted images using Statistical Parametric Mapping (SPM) 12 software. The hippocampal subfield, parahippocampal, entorhinal, perirhinal, precuneus, inferior parietal lobule, and retrosplenial cortical volumes of interest (VOI) were ICV-corrected using the residuals method. This method was originally described by (Mathalon et al., 1993) and it aims to remove the ICV-VOI relationship. The residuals method obtains estimates of the ICV-VOI regression model parameters based on information solely from the control group, and these estimates are then used to compute residuals for the entire dataset (O’Brien et al., 2011). The assumption behind this method is that the regression slope β is representative of a non-pathological relationship between VOI and ICV (Voevodskaya, 2014). Thus, in studies where there is suspected brain atrophy (e.g., due to having a family history of dementia and being APOE e4-positive), the VOI-ICV regression line is calculated from controls (i.e., for the current study, controls are individuals who do not have a family history of dementia and who are also APOE-e4 non-carriers). Cortical thickness was not normalized as thickness measures do not scale linearly with head size.

#### 2.4.3. Diffusion-weighted imaging

**Figure 3:**
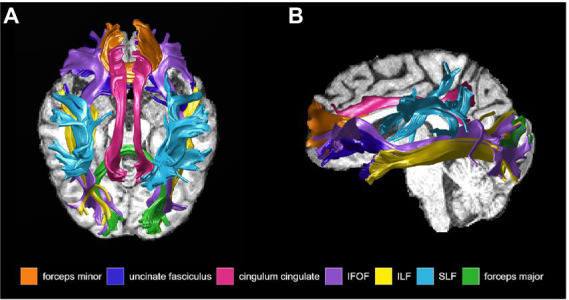
White matter tracts of interest obtained from Automated Fiber Quantification software and used for analysis, shown in **(A)** sagittal, and **(B)** axial view. IFOF, inferior fronto-occipital fasciculus; ILF, inferior longitudinal fasciculus; SLF, superior longitudinal fasciculus.

**Preprocessing**: T1-weighted images for each participant were aligned into AC-PC space to provide a common orientation for brain visualization and tractography. This alignment involved manually defining several anatomical landmarks in the T1 images: the AC (anterior commissure), the PC (posterior commissure), and the mid-sagittal plane. Diffusion-weighted images were preprocessed in FMRIB’s Software Library (FSL) using FMRIB’s Diffusion Toolbox (FDT) for eddy current correction and head motion correction. The preprocessed diffusion-weighted images and the ACPC-aligned T1-weighted images were then input into the automated fiber quantification (AFQ) software (Yeatman et al., 2012). AFQ, a software package implemented in MATLAB, was used to identify the core of 20 major white matter tracts in each subject’s brain and then quantified the tissue properties of voxels near the core of the estimated tracts (Yeatman et al., 2012). The AFQ procedure is based on a combination of the methods described by (Hua et al., 2008) and (Zhang et al., 2008), and can be summarized in 6 steps: (1) *Whole-brain tractography* is done for each subject, where all fibers are tracked with a white matter mask defined as all the voxels with an FA value >0.3; (2) *Fiber tract segmentation* is performed based on the waypoint ROI procedure described in (Wakana et al., 2007). Fibers are assigned as candidates to a specific fiber tract if they pass through two waypoint ROIs that define the central portion of the tract; (3) *Fiber tract refinement* involves scoring each candidate fiber based on its similarity to a standard fiber tract probability map from (Hua et al., 2008). Fibers with high probability scores are retained, thus defining the fiber tract core; (4) *Fiber tract cleaning* filters out stray fibers that deviate substantially from the core of a fiber group represented as a 3-dimensional Gaussian distribution; (5) *Fiber tract clipping* to the central portion that spans between the two defining ROIs for that tract; and finally (6) *Fiber tract quantification* to calculate diffusion measures at 100 equidistant nodes along the trajectory of the fiber group. The diffusion measures are calculated at each node by taking the weighted average of each fiber making up the tract based on its distance from the tract core. Thus, the AFQ pipeline produces a set of “tract profiles” that measure the tissue properties (FA, RD, AxD, and MD) at equidistant sample positions (100 nodes were used for our analysis) from the start to the end of the tract core. There is one profile for each tract and tissue property combination (e.g., FA along the inferior fronto-occipital fasciculus). The 20 fiber tracts identified with AFQ are: bilateral anterior thalamic radiation, bilateral corticospinal tract, bilateral cingulum cingulate, bilateral cingulum hippocampus, callosum forceps minor, callosum forceps major, bilateral inferior fronto-occipital fasciculus, bilateral inferior longitudinal fasciculus, bilateral superior longitudinal fasciculus, bilateral uncinate fasciculus, and bilateral arcuate fasciculus (Hua et al., 2008). The literature has shown that the cingulum bundle, corpus callosum, inferior fronto-occipital fasciculus, inferior longitudinal fasciculus, superior longitudinal fasciculus, and uncinate fasciculus are affected early in AD progression. The corpus callosum, inferior fronto-occipital fasciculus, inferior longitudinal fasciculus, and superior longitudinal fasciculus have also been implicated in non-standard visuomotor transformations. We excluded the cingulum hippocampus which could not be properly tracked and was missing diffusion measures in over 5 subjects. The following 12 tracts of interest were therefore used in the final analysis: left (*n* = 47) and right (*n* = 46) cingulum cingulate (CGC), callosum forceps minor (*n* = 49), callosum forceps major (*n* = 49), left (*n* = 48) and right (*n* = 47) inferior fronto-occipital fasciculus (IFOF), left (*n* = 48) and right (*n* = 49) inferior longitudinal fasciculus (ILF), left (*n* = 49) and right (*n* = 48) superior longitudinal fasciculus (SLF), and left (*n* = 48) and right (*n* = 49) uncinate fasciculus (UF; Figure 3).

### 2.5. Statistical analysis

All statistical analyses were carried out using open-source R software v4.1.0 (R Core Team, 2021). All participant groups were age-balanced, with no statistically significant differences in age observed between individuals with vs. without a family history of dementia (*p* = 0.87), APOE e4 positive vs. e4 negative participants (*p* = 0.64), and female vs. male participants (*p* = 0.82). There were also no statistically significant differences observed between groups on MoCA scores (FH+ vs. FH− *p* = 0.58; e4+ vs. e4− *p* = 0.74; female vs. male *p* = 0.20), years of education (FH+ vs. FH− *p* = 0.57; e4+ vs. e4− *p* = 0.15; female vs. male *p* = 0.54), computer experience (FH+ vs. FH− *p* = 0.36; e4+ vs. e4− *p* = 0.51; female vs. male *p* = 0.46), and touchscreen experience (FH+ vs. FH− *p* = 0.92; e4+ vs. e4− *p* = 0.62; female vs. male *p* = 0.55).

#### 2.5.1. Differences In structural MRI and DTI measures between groups

Three-way ANOVAs were used to compare differences in structural MRI and DTI measures between three groups: (1) individuals with a family history of dementia and individuals without a family history of dementia, (2) females and males, and (3) APOE e4 carriers and non-carriers. Specifically, the neuroimaging measures being compared were (1) grey matter volumes of the hippocampal subfields, parahippocampal cortex, entorhinal cortex, perirhinal cortex, retrosplenial cortex, inferior parietal lobule, and precuneus, (2) grey matter thickness of the parahippocampal cortex, entorhinal cortex, perirhinal cortex, retrosplenial cortex, inferior parietal lobule, and precuneus, and (3) mean diffusivity measures (i.e., FA, MD, RD, AxD) across the white matter tracts of interest. Post-hoc analyses were adjusted for multiple comparisons using the Holm correction method. Corrections were considered statistically significant at an alpha of *p* < 0.05.

#### 2.5.2. Effects of structural MRI and DTI measures on visuomotor performance

Some of the kinematic measures were combined into composite scores to decrease the number of comparisons made in the data analysis, and the procedure was previously described in detail (Rogojin et al., 2019). Briefly, all kinematic measures were standardized using z-scores and composite scores were then created using simple averaging. The timing score was a composite score of RT, MTf, and PV, and the endpoint error score was a composite score of AE and VE. The Cronbach’s alpha values for the timing and endpoint error scores were 0.897 and 0.772, respectively, indicating a high internal consistency.

Multiple regression analyses previously revealed both sex and family history as significant predictors of greater endpoint error scores (indicative of worse visuomotor performance) in the visual feedback reversal condition. The regression analyses also provided preliminary evidence that having an APOE e4 allele was a significant predictor of greater endpoint error scores in the plane-change feedback reversal condition, and greater corrective path lengths (indicative of worse visuomotor performance) in both plane-change conditions. Based on these previous behavioral findings, the current study used multiple linear regression analysis to explore whether visuomotor performance declines are associated with grey and white matter structural neuroimaging measures. The white matter tracts of interest were the cingulum cingulate, callosum forceps minor, callosum forceps major, IFOF, ILF, SLF, and UF. The grey matter regions of interest were the retrosplenial cortex, inferior parietal lobule, and precuneus, all shown to be involved in visually-guided reaching. Regression models were set up with grey or white matter measures as the predictor variable, visuomotor performance measures as the dependent variable, and were controlled for (1) sex and family history in the feedback reversal condition, and (2) APOE e4 status in the two plane-change conditions. The *p*-values were adjusted for multiple comparisons using the Holm correction method and were considered statistically significant at *p* < 0.05. For example, the regression models in the plane-change feedback reversal condition were set up as follows:


$$Y=\beta_{1}X+\beta_{2}Z+\beta_{3}X\cdot Z+\beta_{0}$$

where: Y is the continuous dependent variable,

X is the continuous independent variable,

Z is the dichotomous independent variable,

X*Z is the interaction term calculated as X multiplied by Z,

β_0_ is the intercept,

β_1_ is the effect of X on Y,

β_2_ is the effect of Z on Y, and.

β_3_ is the effect of XZ on Y.

A sample model looking at whether FA in the right ILF is predictive of endpoint error scores in the plane-change feedback reversal condition, moderated by APOE e4 status:


$$\begin{array}{l} Endpointerrorscore\\ =\beta_{1}Right\;ILF+\beta_{2}APOE\;e4\;status\\ +\beta_{3}Right\;ILF\cdot APOE\;e4\;status+\beta_{0} \end{array}$$

If a significant moderated relationship has been identified (i.e., significant interaction X*Z), we used simple slopes analysis to examine the effects of X on Y within the individual levels of Z. A simple slope is the regression of Y on X at a specific value of Z. In this study, all moderator variables (family history of dementia, sex, and APOE e4 status) were binary - therefore, we tested the regression of Y on X at 0 (no family history, female, APOE e4 non-carrier) and 1 (family history of dementia, male, APOE e4 carriers). Simple slopes analysis was used to determine whether either of the slopes differed significantly from zero (the horizontal).

## 3. Results

### 3.1. Grey matter volume and thickness

**Figure 4:**
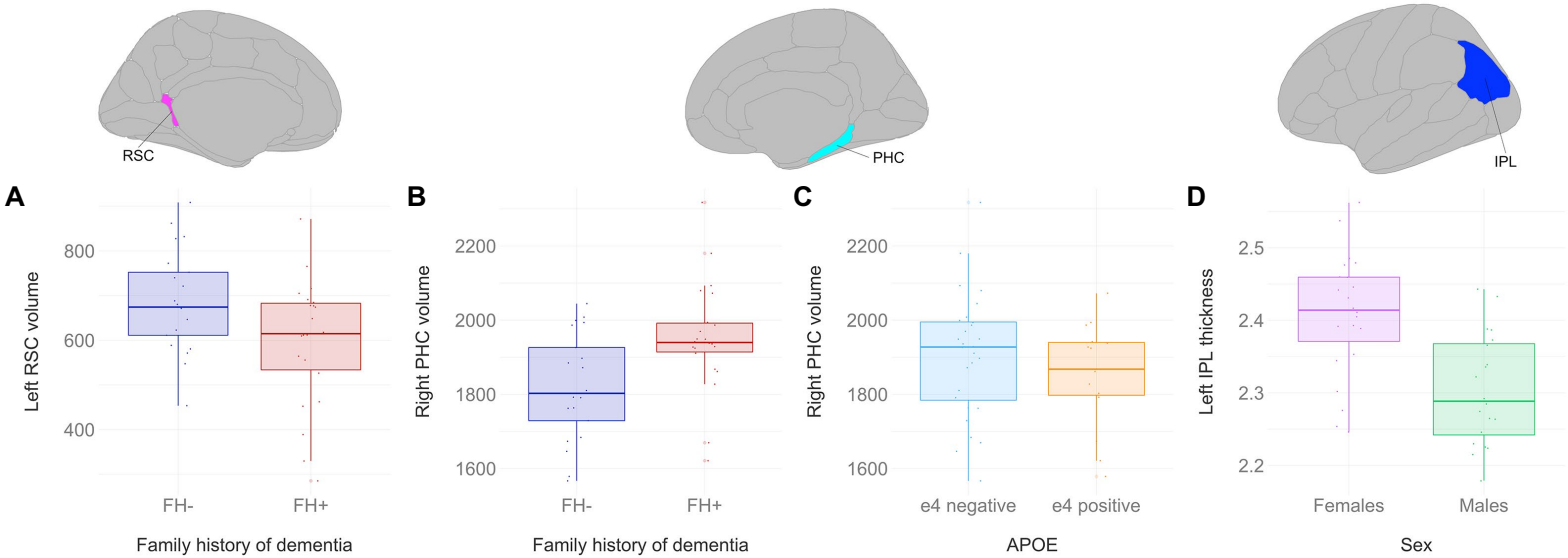
Grey matter volume and thickness differences between participant groups. Significant results shown from three-way ANOVAs. Individuals with a family history of dementia had **(A)** lower left retrosplenial volumes (F_1,43_ = 6.903, *p* < 0.05), but **(B)** greater right parahippocampal volumes compared to individuals without a family history of dementia (F_1,43_ = 14.2647, *p* < 0.01). **(C)** APOE e4 carriers had lower right parahippocampal volumes compared to non-carriers (F_1,43_ = 5.4288, *p* < 0.05). **(D)** Males had lower cortical thickness in the left inferior parietal lobule compared to females (F_1,43_ = 10.1512, *p* < 0.01). Brain figures made with “ggseg” R package (Mowinckel and Vidal-Piñeiro, 2020). RSC, retrosplenial cortex; PHC, parahippocampal cortex; IPL, inferior parietal lobule; FH+, positive family history of dementia; FH−, no family history of dementia; APOE, apolipoprotein E.

There was a significant main effect of family history on retrosplenial cortical volume (Figure 4A), where individuals with a family history of dementia had lower grey matter volumes in the left retrosplenial cortex compared to individuals without a family history of dementia (F_1,43_ = 6.903, *p* < 0.05). There was a significant main effect of family history on parahippocampal cortical volume (Figure 4B), where individuals with a family history of dementia had greater grey matter volumes in the right parahippocampal cortex compared to individuals without a family history of dementia (F_1,43_ = 14.2647, *p* < 0.01). Conversely, there was a significant main effect of APOE status on parahippocampal cortical volume (Figure 4C), where APOE e4 carriers had lower grey matter volumes in the right parahippocampal cortex compared to individuals without an APOE e4 allele (F_1,43_ = 5.4288, *p* < 0.05). Lastly, there was a significant main effect of sex on inferior parietal lobule thickness (Figure 4D), where males had lower cortical thickness in the left inferior parietal lobule compared to females (F_1,43_ = 10.1512, *p* < 0.01). There were no statistically significant differences between (1) positive and negative family history, (2) females and males, and (3) APOE e4 carriers and non-carriers found for grey matter volume or thickness in the hippocampal subfields, entorhinal cortex, and perirhinal cortex.

### 3.2. Relationship between grey matter measures and non-standard visuomotor performance

There were no significant interaction effects between the inferior parietal lobule, precuneus, or restrosplenial cortex and family history, sex, or APOE status in the multiple regression models that were based on previous behavioral findings. Multiple linear regression analyses revealed that presubiculum, subiculum, and parahippocampal volumes, as well as entorhinal cortical thickness were predictors of visuomotor performance in several of the non-standard conditions.

**Figure 5:**
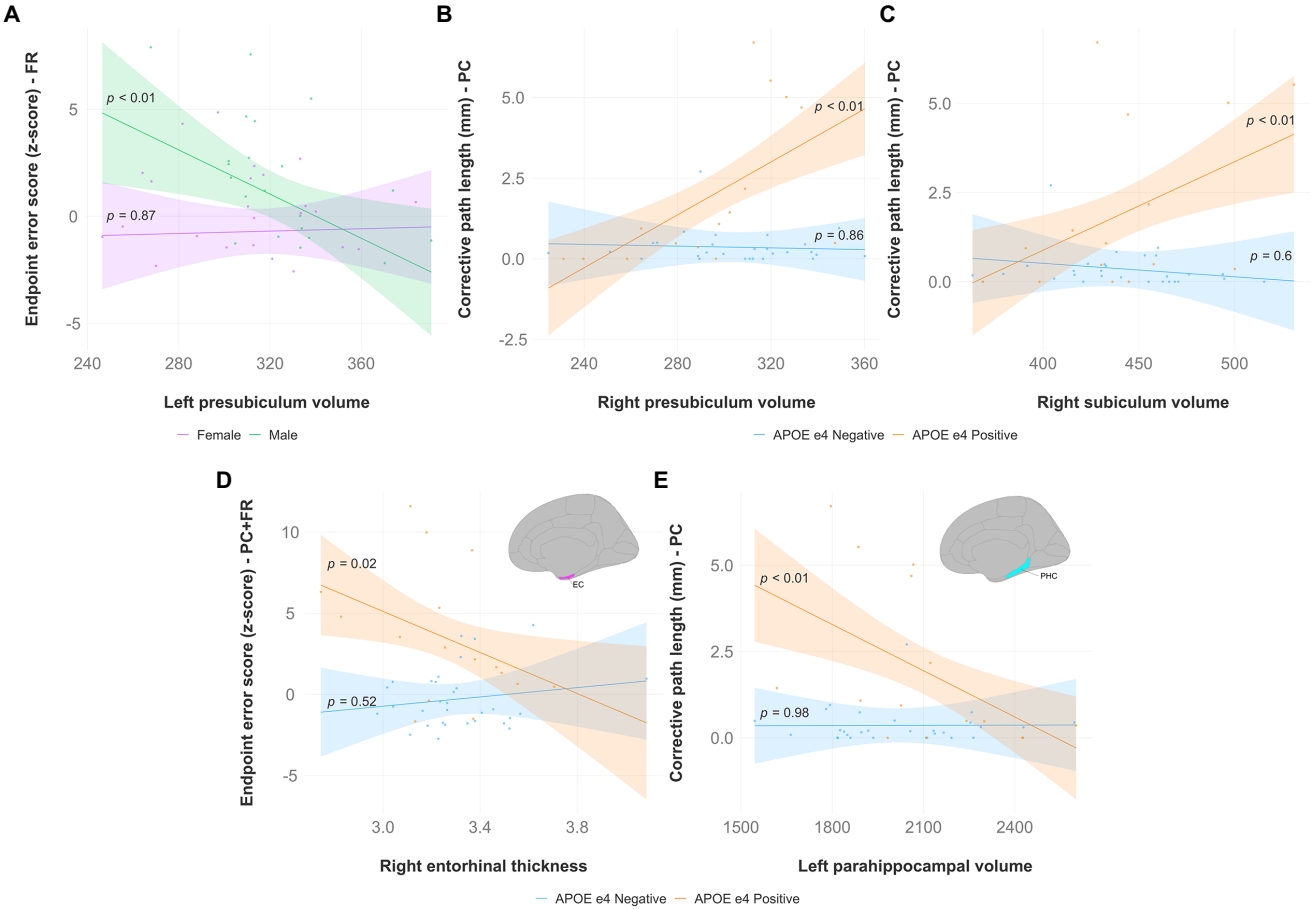
Significant results from hippocampal subfield, entorhinal, and parahippocampal multiple regression and simple slopes analyses. Top panel **(A,B,C)**: Hippocampal subfield volumes predict non-standard visuomotor task performance in the feedback reversal and the plane-change conditions. **(A)** Higher endpoint error scores, reflecting worse visuomotor performance, were associated with smaller left presubiculum volumes (simple slope = −0.06, S.E. = 0.02, *p* < 0.01) only in males. Higher corrective path lengths, reflecting worse visuomotor performance, were associated with **(B)** greater right presubiculum volume (simple slope = 0.04, S.E. = 0.01, *p* < 0.01), and **(C)** greater right subiculum volume (simple slope = 0.02, S.E. = 0.01, *p* < 0.01) only in APOE e4 carriers. Bottom panel **(D,E)**: Entorhinal thickness and parahippocampal volume predict non-standard visuomotor task performance. **(D)** Higher endpoint error scores, reflecting worse visuomotor performance, were associated with lower right entorhinal cortical thickness in the plane-change feedback reversal condition (simple slope = −6.32, S.E. = 2.70, *p* = 0.02) only in APOE e4 carriers. **(E)** Higher corrective path lengths, reflecting worse visuomotor performance, were associated with lower left parahippocampal volume in the plane-change condition (simple slope = −0.004, S.E. = 0.001, *p* < 0.01) only in APOE e4 carriers. Brain figures made with “ggseg” R package (Mowinckel and Vidal-Piñeiro, 2020). EC, entorhinal cortex; PHC, parahippocampal cortex; FR, feedback reversal; PC, plane-change; PC + FR, plane-change feedback reversal; APOE, apolipoprotein E.

**Hippocampal subfields** (Figures 5A–C): There was a significant interaction effect of left presubiculum volume and sex (β = −0.054317, *p* < 0.05) on endpoint error scores in the feedback reversal condition. The results of the regression indicated that the predictors explained 35.8% of variance (R^2^_adj_ = 0.3584, F_5,39_ = 5.915, *p* < 0.001). Simple slopes analysis showed that smaller left presubiculum volume was a significant predictor of greater endpoint error scores only in males (simple slope = −0.06, S.E. = 0.02, *p* < 0.01). There was a significant interaction effect between right presubiculum volume and APOE status (β = 0.042356, *p* < 0.01) on corrective path length in the plane-change condition, where the two predictors explained 43.1% of variance (R^2^_adj_ = 0.4312, F_3,39_ = 11.61, *p* < 0.0001). Simple slopes analysis showed that larger left presubiculum volume was a significant predictor of greater corrective path length only in APOE e4 carriers (simple slope = 0.04, S.E. = 0.01, *p* < 0.01). There was a significant interaction effect between right subiculum volume and APOE status (β = 0.02861, *p* < 0.05) on corrective path length in the plane-change condition, where the two predictors explained 32.5% of variance (R^2^_adj_ = 0.3248, F_3,39_ = 7.736, *p* < 0.001). Simple slopes analysis showed that larger right subiculum volume was a significant predictor of greater endpoint error scores only in APOE e4 carriers (simple slope = 0.02, S.E. = 0.01, *p* < 0.01).

**Entorhinal cortex** (Figure 5D): There was a significant interaction effect of right entorhinal thickness and APOE status (β = −7.759, *p* < 0.05) on endpoint error scores in the plane-change feedback reversal condition. The results of the regression indicated that the predictors explained 34.8% of variance (R^2^_adj_ = 0.3479, F_3,43_ = 9.181, *p* < 0.0001). Simple slopes analysis showed that smaller right entorhinal thickness was a significant predictor of greater endpoint error scores only in APOE e4 carriers (simple slope = −6.32, S.E. = 2.70, *p* = 0.02).

**Parahippocampal cortex** (Figure 5E): There was a significant interaction effect of left parahippocampal volume and APOE status (β = −0.0044851, *p* < 0.05) on corrective path length in the plane-change condition. The results of the regression indicated that the predictors explained 35.2% of variance (R^2^_adj_ = 0.3519, F_3,39_ = 8.601, *p* < 0.001). Simple slopes analysis showed that smaller left parahippocampal volume was a significant predictor of greater corrective path length only in APOE e4 carriers (simple slope = −0.004, S.E. = 0.001, *p* < 0.01). There was also a significant interaction effect of left parahippocampal volume and APOE status (β = −0.02192, *p* < 0.05) on corrective path length in the plane-change feedback reversal condition. The results of the regression indicated that the predictors explained 36.2% of variance (R^2^_adj_ = 0.3616, F_3,43_ = 9.686, *p* < 0.0001), however simple slopes analysis was not significant, indicating that APOE e4 carriers and non-carriers were significantly different from each other but not from zero.

### 3.3. Diffusion measures

There were no statistically significant differences between (1) positive and negative family history, (2) females and males, and (3) APOE e4 carriers and non-carriers found for any of the diffusion measures in the major white matter tracts.

### 3.4. Relationship between diffusion measures and non-standard visuomotor performance

Multiple linear regression analyses revealed that lower FA, higher MD, higher RD, and higher AxD were significant predictors of worse visuomotor performance only in the plane-change feedback reversal condition. Simple slopes analyses revealed that the relationship between the diffusion measures and visuomotor performance was only statistically significant in APOE e4 carriers (summarized in Table 2).

**Table 2:** Summary of DTI findings by tract.

Tract		FA	MD	RD	AxD
Forceps minor	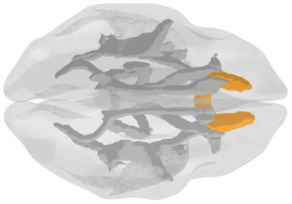	↓	↑	↑	
Forceps major	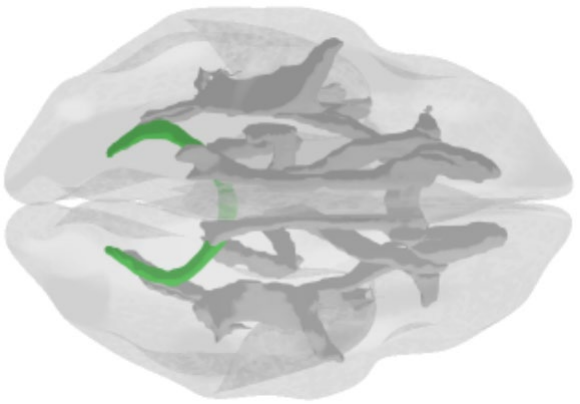		↑		
Cingulum cingulate	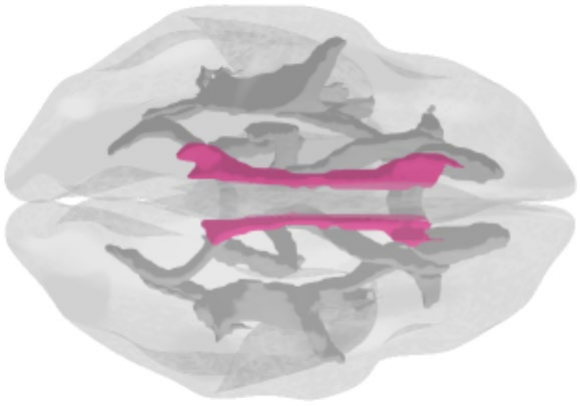		↑ right		↑ right
IFOF	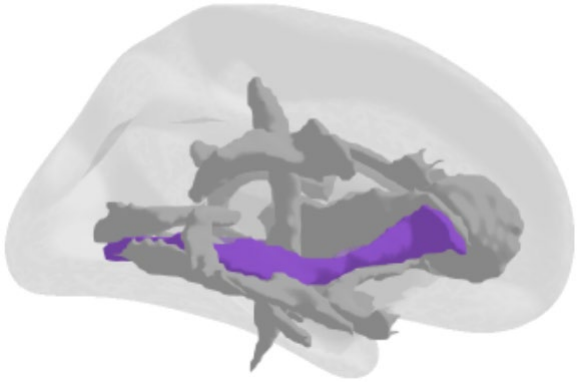	↓ right	↑ bilateral	↑ bilateral	↑ bilateral
ILF	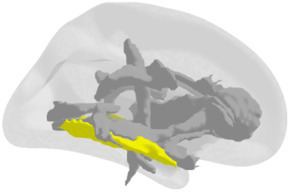	↓ right		↑ right	
SLF	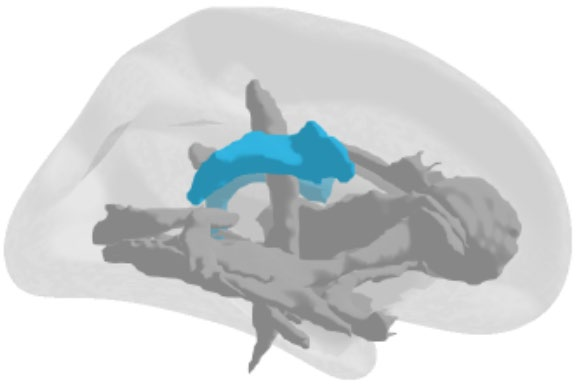				↑ left
UF	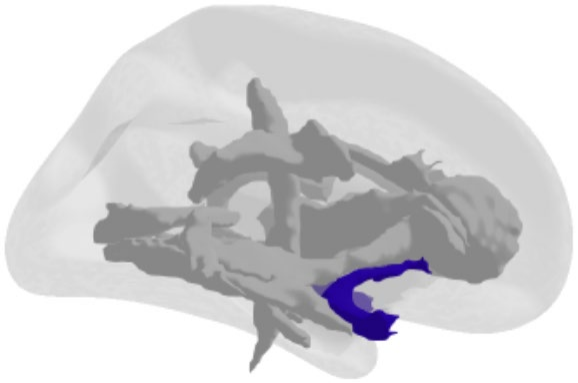				↑ left

In APOE e4 carriers, patterns in diffusion measures shown below (lower FA, higher MD, higher RD, higher AxD) were significant predictors of worse visuomotor performance in the plane-change feedback reversal condition. Brain figures made with “ggseg3d” R package (Mowinckel and Vidal-Piñeiro, 2020). APOE, apolipoprotein E; FA, fractional anisotropy; MD, mean diffusivity; RD, radial diffusivity; AxD, axial diffusivity; IFOF, inferior fronto-occipital fasciculus; ILF, inferior longitudinal fasciculus; SLF, superior longitudinal fasciculus; UF, uncinate fasciculus.

**Figure 6:**
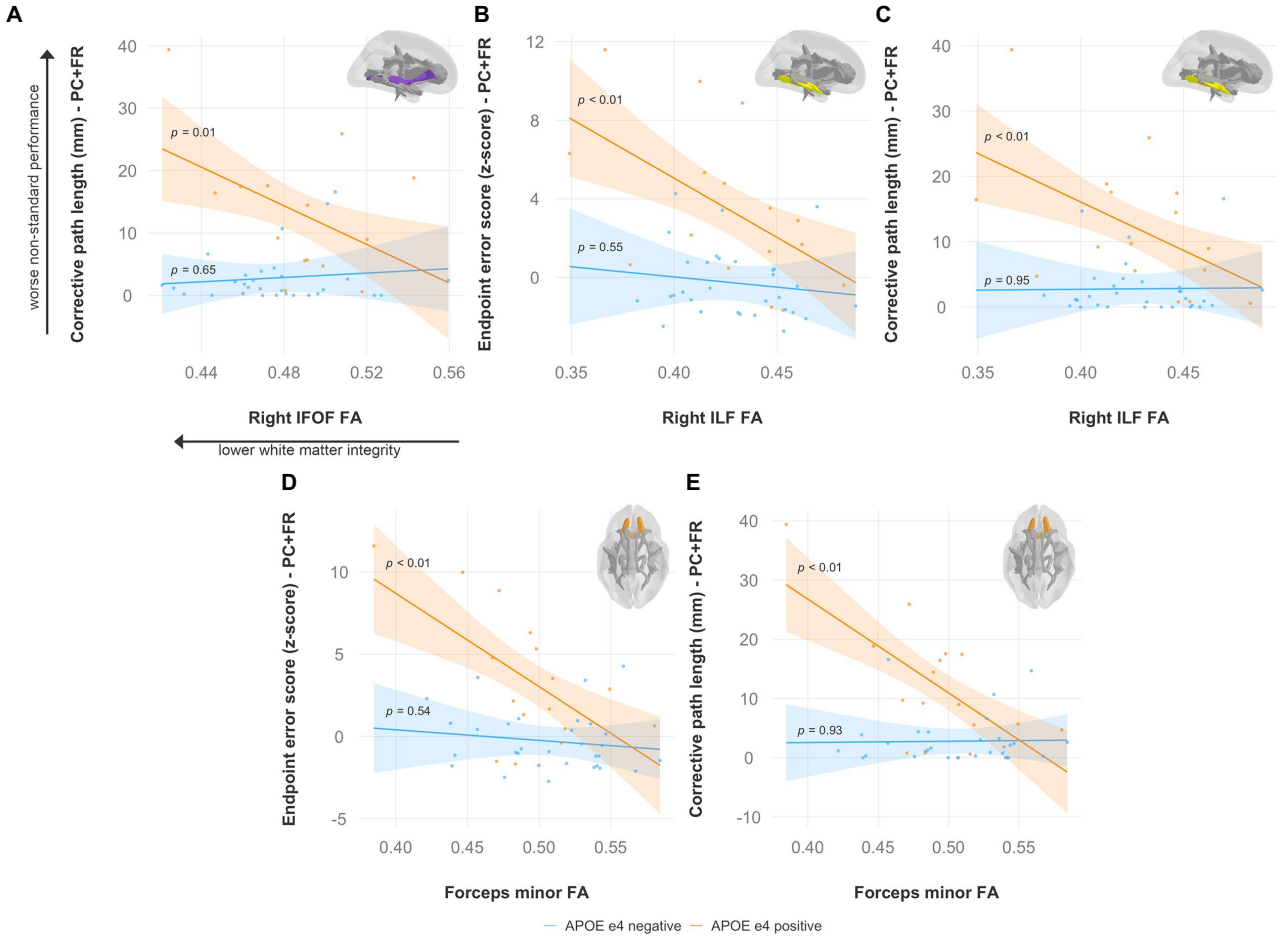
Lower fractional anisotropy predicts worse visuomotor performance (reflected by higher endpoint error scores or corrective path lengths) in the plane-change feedback reversal condition only in APOE e4 carriers in several major white matter tracts, including the **(A)** right IFOF, **(B,C)** right ILF, and **(D,E)** forceps minor. Significant results shown from multiple regression and simple slopes analyses. Brain figures made with “ggseg” R package (Mowinckel and Vidal-Piñeiro, 2020). PC + FR, plane change feedback reversal condition; APOE, apolipoprotein E; FA, fractional anisotropy; IFOF, inferior fronto-occipital fasciculus; ILF, inferior longitudinal fasciculus.

**Figure 7:**
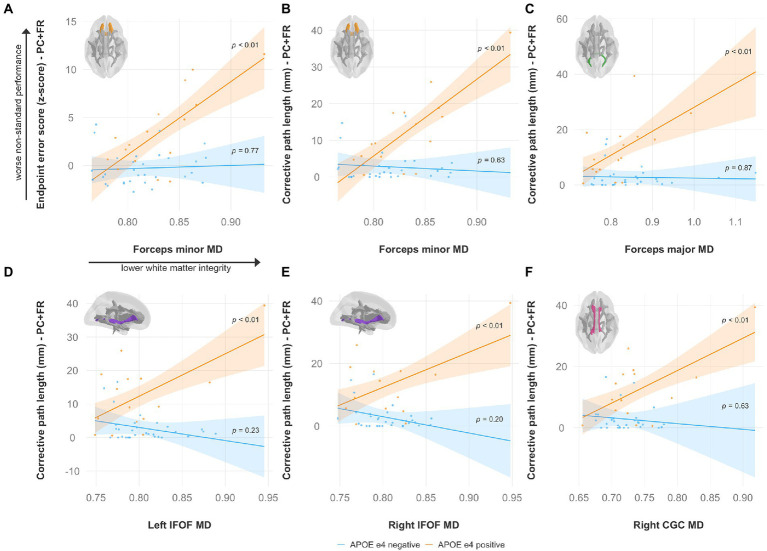
Higher mean diffusivity predicts worse visuomotor performance (reflected by higher endpoint error scores or corrective path lengths) in the plane-change feedback reversal condition only in APOE e4 carriers in several major white matter tracts, including the **(A,B)** forceps minor, **(C)** forceps major, **(D)** left IFOF, **(E)** right IFOF, and **(F)** right CGC. Significant results shown from multiple regression and simple slopes analyses. Brain figures made with “ggseg” R package (Mowinckel and Vidal-Piñeiro, 2020). PC + FR, plane change feedback reversal condition; APOE, apolipoprotein E; MD, mean diffusivity; IFOF, inferior fronto-occipital fasciculus; CGC, cingulum cingulate.

**Figure 8:**
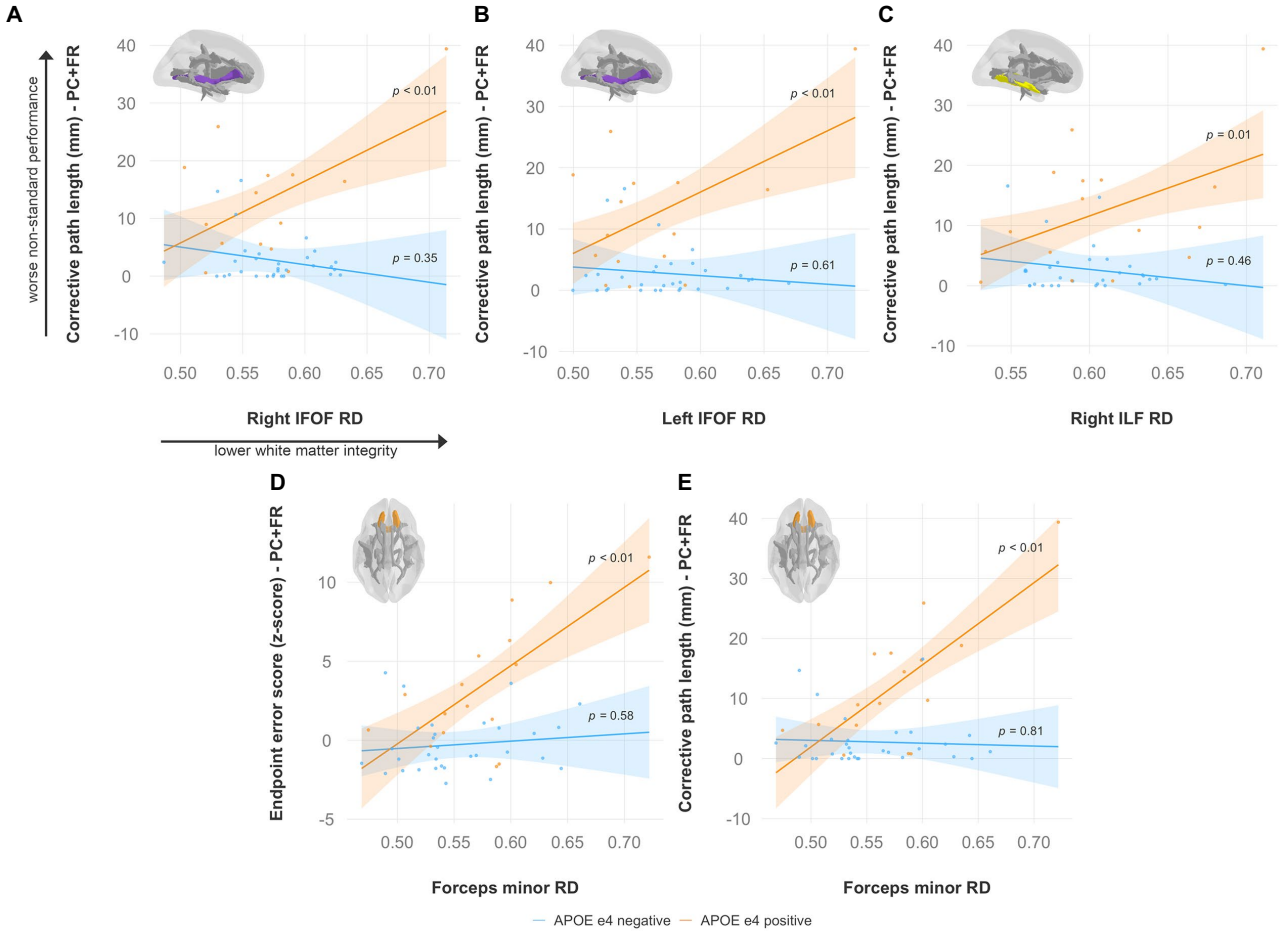
Higher radial diffusivity predicts worse visuomotor performance (reflected by higher endpoint error scores or corrective path lengths) in the plane-change feedback reversal condition only in APOE e4 carriers in several major white matter tracts, including the **(A)** right IFOF, **(B)** left IFOF, **(C)** right ILF, and **(D,E)** forceps minor. Significant results shown from multiple regression and simple slopes analyses. Brain figures made with “ggseg” R package (Mowinckel and Vidal-Piñeiro, 2020). PC + FR, plane change feedback reversal condition; APOE, apolipoprotein E; RD, radial diffusivity; IFOF, inferior fronto-occipital fasciculus; ILF, inferior longitudinal fasciculus.

**Figure 9:**
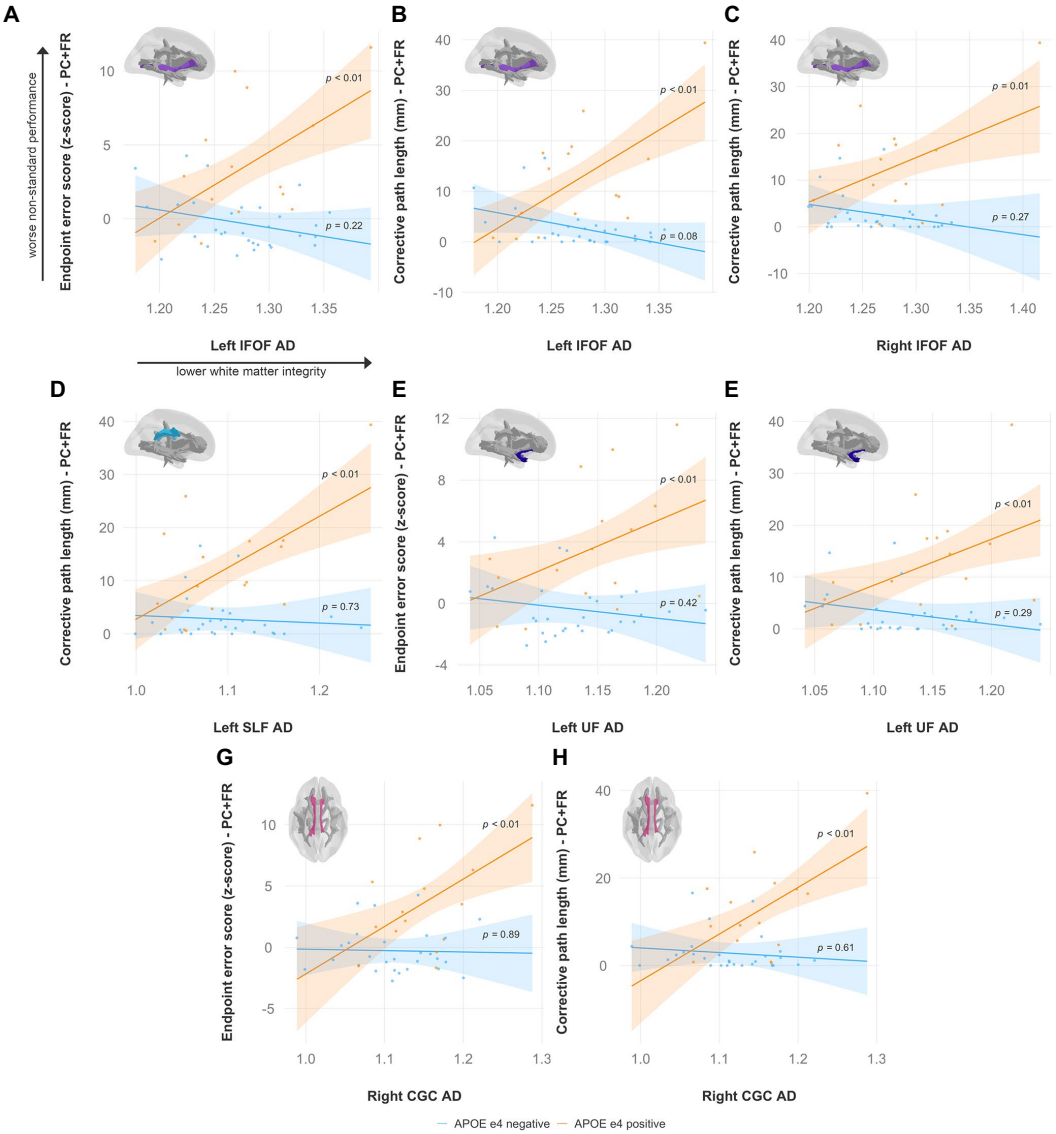
Higher axial diffusivity predicts worse visuomotor performance (reflected by higher endpoint error scores or corrective path lengths) in the plane-change feedback reversal condition only in APOE e4 carriers in several major white matter tracts, including the **(A,B)** left IFOF, **(C)** right IFOF, **(D)** left SLF, **(E,F)** left UF, and **(G,H)** right CGC. Significant results shown from multiple regression and simple slopes analyses. Brain figures made with “ggseg” R package (Mowinckel and Vidal-Piñeiro, 2020). PC + FR, plane change feedback reversal condition; APOE, apolipoprotein E; AxD, axial diffusivity; IFOF, inferior fronto-occipital fasciculus; SLF, superior longitudinal fasciculus; UF, uncinate fasciculus; CGC, cingulum cingulate.

Specifically, lower FA was associated with worse non-standard visuomotor performance in the forceps minor, right IFOF, and right ILF (Figure 6). Higher MD was associated with worse non-standard visuomotor performance in the right cingulum cingulate, forceps major, forceps minor, and bilateral IFOF (Figure 7). Higher RD was associated with worse non-standard visuomotor performance in the forceps minor, bilateral IFOF, and right ILF (Figure 8). Higher AxD was associated with worse non-standard visuomotor performance in the right cingulum cingulate, bilateral IFOF, left SLF, and left UF (Figure 9). Lower FA, higher MD, higher RD, and higher AxD are typically associated with damaged or impaired fiber integrity due to aging or disease pathology (Beaulieu et al., 1996; Davis et al., 2009; Acosta-Cabronero et al., 2010; Salat et al., 2010; Zhang et al., 2010; Bosch et al., 2012; Soares et al., 2013; Doan et al., 2017; Mayo et al., 2019; Bigham et al., 2020). Previous studies in preclinical Alzheimer’s populations found similar patterns of changes in diffusion measure of white matter in the same tracts (Bendlin et al., 2010; Gold et al., 2012; Westlye et al., 2012; Adluru et al., 2014), some of which overlap tracts important for visuomotor integration, namely the SLF, IFOF, ILF, and corpus callosum. Our findings support our hypothesis that white matter integrity declines in these tracts would be associated with declines in non-standard visuomotor integration performance. The statistics for these multiple linear regressions can be found in the Supplementary material.

## 4. Discussion

The main interest of this study was to investigate the grey and white matter neural anatomy underlying impaired visuomotor performance, and to explore the effects of family history of dementia, APOE genotype, and sex. Our key findings suggest that there is a relationship between the APOE e4 genotype and structural brain changes that predicts poorer visuomotor performance. This relationship was demonstrated in our grey matter structural findings, where lower thickness and volume in areas of the medial temporal lobe predicted visuomotor deficits, and then replicated in our diffusion data, where declines in white matter integrity also predicted impaired non-standard visuomotor performance. These visuomotor performance deficits in APOE e4 carriers were detectable using our non-standard visuomotor tasks in the absence of any measurable purely cognitive issues. We also found sex-differences in grey matter thickness and volume in areas of the posterior parietal cortex and hippocampus that are important for visuospatial processing, which may explain the poorer visual feedback reversal task performance we previously reported in males.

### 4.1. Grey matter measures underlying visuomotor deficits in individuals with an increased risk for Alzheimer’s disease

We first examined group differences in grey matter volume and thickness. Individuals with a family history of dementia had greater grey matter volumes in the right parahippocampal cortex compared to those without a family history of dementia. Conversely, APOE e4 carriers had lower grey matter volumes in the right parahippocampal cortex compared to individuals without an APOE e4 allele. Individuals with a family history of dementia also had lower grey matter volumes in the left retrosplenial cortex. Our main interest, however, was exploring whether structural differences in the inferior parietal lobule, precuneus, retrosplenial cortex, and areas of the medial temporal lobe may underlie visuomotor integration deficits in individuals at an increased risk for AD. Our results for hippocampal subfield volumes were surprising. We found that poorer non-standard visuomotor performance was associated with greater grey matter volumes in the right presubiculum and right subiculum. This relationship was only significant for APOE e4 carriers and is contrary to our hypothesis that atrophy within the hippocampus, one of the medial temporal lobe regions involved in visuomotor control (Tankus and Fried, 2012), may be predictive of worse visuomotor task performance. However, it should be noted that the automated hippocampal subfield segmentation module in FreeSurfer has not been validated against histological annotations within the same subjects, nor against manual segmentation on the same MRI scans (Wisse et al., 2021) caution against using 1 × 1 × 1 mm^3^ T1 scans for automatic segmentation of the hippocampus as the subfields are difficult to discern at this resolution. Studies that used automatic hippocampal segmentation have been shown to report contradictory and sometimes neurobiologically unexpected results (Haukvik et al., 2018; Wisse et al., 2021). Indeed (Iglesias et al., 2015), themselves mention that the volumetric results from individual subfields automatically segmented in FreeSurfer using only standard resolution (1 mm) T1 data must be interpreted with caution.

In APOE e4 carriers, a lower left parahippocampal volume and lower right entorhinal thickness were associated with worse non-standard visuomotor task performance. As mentioned previously, the hippocampal, parahippocampal, entorhinal, and retrosplenial cortices have been implicated in visuospatial processing and visuomotor control (Vann et al., 2009; Tankus and Fried, 2012). In addition to prominent memory declines, AD patients also show impairments in visuospatial processing and skilled eye-hand coordination performance (Ghilardi et al., 1999; Tippett and Sergio, 2006; Tippett et al., 2007). Apraxia, a disorder of skilled movements, is seen in some AD patients and is included as one of the criteria for AD (Lesourd et al., 2013; Dubois et al., 2014; Smits et al., 2014). Regions of the medial temporal lobe, including the hippocampus (subiculum, presubiculum, and parasubiculum) and parahippocampus, are interconnected with the parietal cortex *via* the retrosplenial cortex, which itself is involved in episodic memory, navigation, and translation between different frames of reference (Vann et al., 2009). Thus, early dysfunction in not only medial temporal regions but also in regions involved in visuomotor control could prove to be useful for understanding the underlying pathophysiology of AD and predicting AD risk.

Some of our findings showed increased volumes in regions of medial temporal lobe in individuals at an increased risk for AD, both in group differences (family history versus no family history of dementia) as well as associated with non-standard visuomotor deficits. Converging evidence suggests that by the time clinical impairment is detectable in AD, extensive neurodegeneration has already taken place (Morris, 2005). However, the relationship between AD pathology and brain atrophy in preclinical AD is still under debate (Chételat et al., 2013). Grey matter atrophy has been reported in cognitively healthy individuals positive for amyloid pathology (Fagan et al., 2009; Mormino et al., 2009; Villeneuve et al., 2014) and tau pathology (Wang et al., 2016; Scott et al., 2020; Xie et al., 2020), family history of dementia (Honea et al., 2010, 2011; Mosconi et al., 2014), and the APOE e4 allele (O’Dwyer et al., 2012; Haller et al., 2017; Veldsman et al., 2021). Conversely, other studies have reported increased volumes in areas of the medial temporal lobe in preclinical AD (Chételat, Villemagne, Pike, et al., 2010; Gispert et al., 2015). Therefore, the changes in grey matter volume in preclinical AD may not necessarily be linear.

### 4.2. Pattern of white matter integrity declines are generally consistent with the literature and predict visuomotor deficits in APOE e4 carriers

Studies examining white matter diffusion characteristics have demonstrated that white matter may be independently affected from amyloid pathology (Rabin et al., 2019) and grey matter atrophy (Amlien and Fjell, 2014) early in the pathological process, emphasizing the importance of investigating white matter changes underlying disease progression. White matter microstructural reductions have been shown in cognitively healthy individuals at an increased risk for AD (due to family history of dementia and having an APOE e4 allele) in the absence of medial temporal lobe atrophy (Gold et al., 2010). In the current study, we found no differences in white matter diffusion measures between groups (i.e., family history status, sex, or APOE e4 status). While there were no group differences in white matter integrity alone, we did observe that in APOE e4 carriers, diffusion measures of several major white matter tracts were predictive of non-standard visuomotor performance. Specifically, we found that lower FA (in the forceps minor, right IFOF, and right ILF), higher MD (in the right cingulum cingulate, forceps major, forceps minor, and bilateral IFOF), higher RD (in the forceps minor, right ILF, and bilateral IFOF), and higher AxD (in the cingulum cingulate, bilateral IFOF, left SLF, and left UF) were predictive of worse performance in the most cognitively demanding non-standard visuomotor condition (plane-change feedback reversal). Our findings are consistent with the literature, where white matter alterations have previously been detected in cognitively healthy individuals with the APOE e4 allele, with similar patterns of reduced white matter microstructure (i.e., reduced FA, and increased MD, RD, AxD) in some of the same tracts affected in our results, including the cingulum cingulate, forceps minor, IFOF, SLF, and ILF (Westlye et al., 2012; Adluru et al., 2014; Cavedo et al., 2018; Operto et al., 2018).

Our findings suggest that lower white matter integrity in several major tracts in the brain may underlie the non-standard visuomotor deficits we previously demonstrated in APOE e4 carriers compared to non-carriers. In the brain, the APOE protein plays a central role in cholesterol transport (Mahley and Rall, 2000), which is essential for axonal growth as well as synaptic formation and remodeling (Liu et al., 2013). APOE may affect white matter microstructure due to the e4 isoform having a reduced capacity of keeping cholesterol homeostasis in the brain, leading to disruptions of the myelin sheath (Operto et al., 2018). Vulnerability to the degradation of white matter microstructure may therefore be particularly high or accelerated in APOE e4 carriers. The resultant myelin disruption and axonal injury in tracts projecting between brain regions necessary for non-standard visuomotor integration, a task that heavily relies on large scale frontoparietal networks, may impact effective communication across these areas and be reflected as visuomotor deficits. Indeed, the white matter tracts in which lower white matter integrity was associated with visuomotor performance declines are some of the same white matter tracts that comprise the frontoparietal network, including the SLF, IFOF, and ILF (de Schotten et al., 2011; Rodríguez-Herreros et al., 2015; Budisavljevic et al., 2017; Waller et al., 2017; Herbet et al., 2018).

### 4.3. Grey matter structure underlying sex-differences in non-standard visuomotor performance

We previously showed that sex was a significant predictor of visuomotor performance in the feedback reversal condition, where males had greater endpoint error scores compared to females after controlling for family history and APOE status (Rogojin et al., 2019). In the current study, we found that males had lower left inferior parietal lobule thickness compared to females. While the right inferior parietal lobule in particular appears to be involved in non-standard visuomotor transformations involving a visual feedback reversal (Gorbet and Sergio, 2016) and is a crucial node in the frontoparietal network needed for maintaining spatial attention on current task goals (Singh-Curry and Husain, 2009), the left inferior parietal lobule has been shown to be important for visually-guided reaches as well. There was bilateral activation in the inferior parietal lobule during a reaching task, and reaching to visual targets compared to proprioceptive targets incurred more activity in the left inferior parietal lobule (Bernier and Grafton, 2010). Furthermore, the feedback reversal condition used in the current study is a version of visuomotor adaptation, and the left inferior parietal lobule is critical for successful visuomotor adaptation (Mutha et al., 2011; Crottaz-Herbette et al., 2014). It is important to note that the lower left inferior lobule thickness found in males may just be a general structural sex-difference rather than a result of age-related atrophy, as others have previously demonstrated greater cortical thickness in the inferior parietal lobules of adult females compared to males (Sowell et al., 2007). We found that having a lower left presubiculum volume was also associated with greater endpoint error scores in the visual feedback reversal condition only in males. The presubiculum of the hippocampus has extensive interconnections with the inferior parietal lobule (Rockland and Van Hoesen, 1999; Ding et al., 2000; Clower et al., 2001), and the hippocampus itself has been implicated in visuomotor coordination (Tankus and Fried, 2012).

### 4.4. Limitations and future directions

One of the limitations of the current study was that hippocampal subfield segmentation was obtained from T1-weighted MRI scans, which have limited tissue contrast of hippocampal structures (Wisse et al., 2021). The number of APOE e4 negative participants in the current study was also greater than the number of APOE e4 carriers. Despite this limitation, the preliminary findings on the relationship between APOE genotype, structural measures, and non-standard visuomotor performance indicate a potential mechanism underlying previous behavioral findings of visuomotor impairments in APOE e4 carriers and represent an important starting point for subsequent investigations. Another limitation is that this study was cross-sectional in nature. It would be interesting to conduct a longitudinal study to investigate if participants demonstrating visuomotor deficits prior to cognitive decline are more likely to develop mild cognitive impairment or dementia later in life. Preclinical AD has been hypothesized to begin decades before the onset of clinical symptoms. One proposed sequence of events leading to dementia suggests that disruptions in the default mode network may precede neurodegeneration (Sheline and Raichle, 2013). Several of the core nodes of the default mode network (Parvizi et al., 2006; Sperling et al., 2010; Márquez and Yassa, 2019) overlap with brain areas shown to be involved in non-standard visuomotor integration (Gorbet and Sergio, 2016, 2018). Future work could therefore investigate whether there are differences in default mode network connectivity between groups based on family history, genetic risk, or sex, and whether disruptions in functional connectivity underlie visuomotor deficits.

### 4.5. General conclusion

Our results suggest that a visuomotor task may have potential for detecting the early effects of the APOE e4 allele on white matter microstructure and medial temporal lobe grey matter early in dementia progression, as the white matter and grey matter declines observed here were associated with visuomotor difficulties only in e4 carriers. Importantly, the visuomotor deficits were seen prior to group differences in volumetric and diffusion measures due to genetic risk, and *in advance of any cognitive deficits*. We also found structural grey matter differences in the inferior parietal lobule that may underlie the sex-related differences we have previously demonstrated in non-standard visuomotor performance.

## Data availability statement

The raw data supporting the conclusions of this article will be made available by the authors, without undue reservation.

## Ethics statement

The studies involving human participants were reviewed and approved by Human Participants Review Sub-Committee of York University’s Ethics Review Board. The patients/participants provided their written informed consent to participate in this study.

## Author contributions

AR: conceptualization, methodology, software, formal analysis, investigation, data curation, writing – original draft, writing – review and editing, and visualization. DG: conceptualization, methodology, software, formal analysis, resources, data curation, and writing – review and editing. KH: conceptualization, methodology, and investigation. LS: conceptualization, methodology, formal analysis, resources, writing – review and editing, supervision, project administration, and funding acquisition. All authors contributed to the article and approved the submitted version.

## Funding

This work was supported by a Canadian Institutes of Health Research operating grant (grant number MOP-125915 to LS).

## Conflict of interest

The authors declare that the research was conducted in the absence of any commercial or financial relationships that could be construed as a potential conflict of interest.

## Publisher’s note

All claims expressed in this article are solely those of the authors and do not necessarily represent those of their affiliated organizations, or those of the publisher, the editors and the reviewers. Any product that may be evaluated in this article, or claim that may be made by its manufacturer, is not guaranteed or endorsed by the publisher.
