# Supplementary material for: Differences in structural MRI and diffusion tensor imaging underlie visuomotor performance declines in older adults with an increased risk for Alzheimer’s disease
Source: Front Aging Neurosci. 2023 Jan 12;14:1054516. doi: 10.3389/fnagi.2022.1054516 (PMC9877535; doi:10.3389/fnagi.2022.1054516)
Supplement: Supplementary file 1 [file Table_1.docx]

##### Supplementary Table 1. Multiple linear regression models used to explore whether visuomotor performance declines are associated with white matter structural neuroimaging measures. Regression models were set up with white matter measures (FA, MD, RD, AxD) as the predictor variable, APOE e4 status as the moderator variable, and visuomotor performance measures (EE score and CPL) in the plane change feedback reversal condition as the outcome variable. Models with significant interactions are shown.

| **Outcome** | **Tract** | **Predictor** | **Estimate** | **S.E.** | **t** | **Unadjusted *p*** | **Adjusted *p*** |
| --- | --- | --- | --- | --- | --- | --- | --- |
| EE Score | Forceps minor | Intercept | 17.19 | 4.42 | 3.89 | 0.00034 |  |
|  |  | FA | -31.60 | 8.86 | -3.57 | 0.00090 | 0.00271 |
|  |  | APOE | 28.41 | 8.84 | 3.21 | 0.00248 | 0.00497 |
|  |  | FA*APOE | -50.30 | 17.72 | -2.84 | 0.00689 | 0.00689 |
| R^2^_adj_ = 0.4602, F_3,43_ = 14.07, *p* = 0.000001574 | | | | | | | |
| APOE e4 simple slope = -56.75, S.E. = 14.33, *p* < 0.01 | | | | | | | |
| EE Score | Forceps minor | Intercept | -31.44 | 7.79 | -4.04 | 0.00022 |  |
|  |  | MD | 39.80 | 9.44 | 4.22 | 0.00013 | 0.00038 |
|  |  | APOE | -56.72 | 15.58 | -3.64 | 0.00073 | 0.00073 |
|  |  | MD*APOE | 72.72 | 18.89 | 3.85 | 0.00039 | 0.00058 |
| R^2^_adj_ = 0.5444, F_3,43_ = 19.32, *p* = 0.00000004399 | | | | | | | |
| APOE e4 simple slope = 76.16, S.E. = 14.69, *p* < 0.01 | | | | | | | |
| EE Score | Forceps minor | Intercept | -13.94 | 3.79 | -3.68 | 0.00065 |  |
|  |  | RD | 27.11 | 6.66 | 4.07 | 0.00020 | 0.00059 |
|  |  | APOE | -22.19 | 7.58 | -2.93 | 0.00542 | 0.00542 |
|  |  | RD*APOE | 44.96 | 13.31 | 3.38 | 0.00157 | 0.00235 |
| R^2^_adj_ = 0.5149, F_3,43_ = 17.28, *p* = 0.0000001652 | | | | | | | |
| APOE e4 simple slope = 49.59, S.E. = 10.48, *p* < 0.01 | | | | | | | |
| CPL | Forceps minor | Intercept | 45.94 | 10.56 | 4.35 | 0.00008 |  |
|  |  | FA | -78.17 | 21.17 | -3.69 | 0.00062 | 0.00092 |
|  |  | APOE | 88.46 | 21.13 | 4.19 | 0.00014 | 0.00041 |
|  |  | FA*APOE | -160.60 | 42.35 | -3.79 | 0.00046 | 0.00092 |
| R^2^_adj_ = 0.5054, F_3,43_ = 16.67, *p* = 0.0000002487 | | | | | | | |
| APOE e4 simple slope = -158.47, S.E. = 34.25, p < 0.01 | | | | | | | |
| CPL | Forceps minor | Intercept | -73.99 | 18.07 | -4.09 | 0.00018 |  |
|  |  | MD | 97.93 | 21.91 | 4.47 | 0.00006 | 0.00006 |
|  |  | APOE | -175.48 | 36.14 | -4.86 | 0.00002 | 0.00002 |
|  |  | MD*APOE | 222.80 | 43.82 | 5.09 | 0.00001 | 0.00002 |
| R^2^_adj_ = 0.6066, F_3,43_ = 24.64, *p* = 0.000000001956 | | | | | | | |
| APOE e4 simple slope = 209.33, S.E. = 34.07, *p* < 0.01 | | | | | | | |
| CPL | Forceps minor | Intercept | -30.51 | 8.93 | -3.42 | 0.00139 |  |
|  |  | RD | 66.01 | 15.68 | 4.21 | 0.00013 | 0.00019 |
|  |  | APOE | -71.88 | 17.85 | -4.03 | 0.00023 | 0.00023 |
|  |  | RD*APOE | 141.56 | 31.37 | 4.51 | 0.00005 | 0.00015 |
| R^2^_adj_ = 0.5683, F_3,43_ = 21.18, *p* = 0.00000001411 | | | | | | | |
| APOE e4 simple slope = 136.79, S.E. = 24.68, *p* < 0.01 | | | | | | | |
| CPL | Forceps major | Intercept | -26.96 | 11.19 | -2.41 | 0.02030 |  |
|  |  | MD | 42.21 | 13.52 | 3.12 | 0.00321 | 0.00482 |
|  |  | APOE | -63.20 | 22.37 | -2.83 | 0.00715 | 0.00715 |
|  |  | MD*APOE | 88.78 | 27.04 | 3.28 | 0.00205 | 0.00482 |
| R^2^_adj_ = 0.4346, F_3,43_ = 12.79, *p* = 0.000004156 | | | | | | | |
| APOE e4 simple slope = 86.60, S.E. = 23.72, *p* < 0.01 | | | | | | | |
| EE Score | Right CGC | Intercept | -19.91 | 8.45 | -2.36 | 0.02340 |  |
|  |  | AxD | 18.76 | 7.41 | 2.53 | 0.01540 | 0.01770 |
|  |  | APOE | -41.81 | 16.90 | -2.48 | 0.01770 | 0.01770 |
|  |  | AxD*APOE | 39.80 | 14.82 | 2.69 | 0.01050 | 0.01770 |
| R^2^_adj_ = 0.4183, F_3,40_ = 11.31, *p* = 0.00001659 | | | | | | | |
| APOE e4 simple slope = 38.66, S.E. = 12.19, *p* < 0.01 | | | | | | | |
| CPL | Right CGC | Intercept | -25.00 | 16.70 | -1.50 | 0.14220 |  |
|  |  | MD | 43.78 | 22.91 | 1.91 | 0.06320 | 0.06320 |
|  |  | APOE | -82.42 | 33.39 | -2.47 | 0.01800 | 0.02700 |
|  |  | MD*APOE | 124.68 | 45.83 | 2.72 | 0.00960 | 0.02700 |
| R^2^_adj_ = 0.4897, F_3,40_ = 14.75, *p* = 0.000001286 | | | | | | | |
| APOE e4 simple slope = 106.12, S.E. = 25.49, *p* < 0.01 | | | | | | | |
| CPL | Right CGC | Intercept | -47.77 | 20.59 | -2.32 | 0.02549 |  |
|  |  | AxD | 48.05 | 18.06 | 2.66 | 0.01118 | 0.01118 |
|  |  | APOE | -124.80 | 41.17 | -3.03 | 0.00426 | 0.00639 |
|  |  | AxD*APOE | 117.30 | 36.12 | 3.25 | 0.00236 | 0.00639 |
| R^2^_adj_ = 0.4474, F_3,40_ = 12.61, *p* = 0.000006087 | | | | | | | |
| APOE e4 simple slope = 106.70, S.E. = 29.70, *p* < 0.01 | | | | | | | |
| EE Score | Left IFOF | Intercept | -19.29 | 10.07 | -1.92 | 0.06223 |  |
|  |  | AxD | 16.35 | 7.89 | 2.07 | 0.04449 | 0.04445 |
|  |  | APOE | -68.60 | 20.13 | -3.41 | 0.00146 | 0.00219 |
|  |  | AxD*APOE | 56.71 | 15.78 | 3.59 | 0.00085 | 0.00219 |
| R^2^_adj_ = 0.4325, F_3,42_ = 12.43, *p* = 0.000005912 | | | | | | | |
| APOE e4 simple slope = 44.70, S.E. = 12.49, *p* < 0.01 | | | | | | | |
| CPL | Left IFOF | Intercept | -27.39 | 17.72 | -1.55 | 0.12964 |  |
|  |  | MD | 43.80 | 22.02 | 1.99 | 0.05324 | 0.05324 |
|  |  | APOE | -123.46 | 35.43 | -3.48 | 0.00117 | 0.00175 |
|  |  | MD*APOE | 165.93 | 44.04 | 3.77 | 0.00051 | 0.00152 |
| R^2^_adj_ = 0.4872, F_3,42_ = 15.25, *p* = 0.0000007371 | | | | | | | |
| APOE e4 simple slope = 126.76, S.E. = 30.43, *p* < 0.01 | | | | | | | |
| CPL | Left IFOF | Intercept | -16.57 | 11.39 | -1.46 | 0.15316 |  |
|  |  | RD | 42.97 | 20.00 | 2.15 | 0.03749 | 0.03749 |
|  |  | APOE | -54.78 | 22.78 | -2.41 | 0.02067 | 0.03101 |
|  |  | RD*APOE | 114.04 | 40.00 | 2.85 | 0.00673 | 0.02019 |
| R^2^_adj_ = 0.4236, F_3,42_ = 12.02, *p* = 0.000008145 | | | | | | | |
| APOE e4 simple slope = 99.99, S.E. = 29.01, *p* < 0.01 | | | | | | | |
| CPL | Left IFOF | Intercept | -49.43 | 23.42 | -2.11 | 0.04080 |  |
|  |  | AxD | 44.72 | 18.36 | 2.44 | 0.01920 | 0.01920 |
|  |  | APOE | -206.24 | 46.85 | -4.40 | 0.00007 | 0.00011 |
|  |  | AxD*APOE | 169.32 | 36.72 | 4.61 | 0.00004 | 0.00011 |
| R^2^_adj_ = 0.5198, F_3,42_ = 17.23, *p* = 0.0000001911 | | | | | | | |
| APOE e4 simple slope = 129.37, S.E. = 29.06, *p* < 0.01 | | | | | | | |
| CPL | Right IFOF | Intercept | 41.57 | 16.58 | 2.51 | 0.01625 |  |
|  |  | FA | -68.66 | 34.23 | -2.01 | 0.05146 | 0.05146 |
|  |  | APOE | 94.14 | 33.17 | 2.84 | 0.00702 | 0.02106 |
|  |  | FA*APOE | -172.21 | 68.45 | -2.52 | 0.01589 | 0.03178 |
| R^2^_adj_ = 0.4094, F_3,41_ = 11.17, *p* = 0.00001727 | | | | | | | |
| APOE e4 simple slope = -154.77, S.E. = 57.00, *p* = 0.01 | | | | | | | |
| CPL | Right IFOF | Intercept | -16.66 | 20.80 | -0.80 | 0.42761 |  |
|  |  | MD | 30.40 | 25.80 | 1.18 | 0.24549 | 0.24549 |
|  |  | APOE | -122.55 | 41.59 | -2.95 | 0.00528 | 0.00792 |
|  |  | MD*APOE | 164.96 | 51.61 | 3.20 | 0.00268 | 0.00792 |
| R^2^_adj_ = 0.4717, F_3,41_ = 14.09, *p* = 0.000001858 | | | | | | | |
| APOE e4 simple slope = 112.88, S.E. = 33.18, *p* < 0.01 | | | | | | | |
| CPL | Right IFOF | Intercept | -13.76 | 12.94 | -1.06 | 0.29385 |  |
|  |  | RD | 38.32 | 22.57 | 1.70 | 0.09706 | 0.09706 |
|  |  | APOE | -68.10 | 25.89 | -2.63 | 0.01195 | 0.01794 |
|  |  | RD*APOE | 137.62 | 45.13 | 3.05 | 0.00401 | 0.01203 |
| R^2^_adj_ = 0.4648, F_3,41_ = 13.74, *p* = 0.000002407 | | | | | | | |
| APOE e4 simple slope = 107.13, S.E. = 31.27, *p* < 0.01 | | | | | | | |
| CPL | Right IFOF | Intercept | -32.43 | 28.96 | -1.12 | 0.26925 |  |
|  |  | AxD | 31.24 | 22.71 | 1.38 | 0.17628 | 0.17628 |
|  |  | APOE | -152.27 | 57.92 | -2.63 | 0.01200 | 0.01800 |
|  |  | AxD*APOE | 127.33 | 45.41 | 2.80 | 0.00768 | 0.01800 |
| R^2^_adj_ = 0.4224, F_3,41_ = 11.72, *p* = 0.00001109 | | | | | | | |
| APOE e4 simple slope = 94.91, S.E. = 34.80, *p* = 0.01 | | | | | | | |
| EE Score | Right ILF | Intercept | 16.64 | 5.29 | 3.14 | 0.00302 |  |
|  |  | FA | -35.26 | 12.34 | -2.86 | 0.00657 | 0.01971 |
|  |  | APOE | 24.94 | 10.59 | 2.36 | 0.02314 | 0.04628 |
|  |  | FA*APOE | -49.78 | 24.69 | -2.02 | 0.05002 | 0.05000 |
| R^2^_adj_ = 0.4212, F_3,43_ = 14.07, *p* = 0.000006795 | | | | | | | |
| APOE e4 simple slope = -60.15, S.E. = 17.56, *p* < 0.01 | | | | | | | |
| CPL | Right ILF | Intercept | 38.52 | 13.31 | 2.89 | 0.00596 |  |
|  |  | FA | -72.82 | 31.04 | -2.35 | 0.02365 | 0.03880 |
|  |  | APOE | 73.67 | 26.63 | 2.77 | 0.00831 | 0.02493 |
|  |  | FA*APOE | -150.79 | 62.08 | -2.43 | 0.01940 | 0.03880 |
| R^2^_adj_ = 0.4129, F_3,43_ = 11.79, *p* = 0.000009155 | | | | | | | |
| APOE e4 simple slope = -148.21, S.E. = 44.16, *p* < 0.01 | | | | | | | |
| CPL | Right ILF | Intercept | -12.50 | 14.42 | -0.87 | 0.39050 |  |
|  |  | RD | 32.76 | 23.92 | 1.37 | 0.17800 | 0.17800 |
|  |  | APOE | -63.00 | 28.83 | -2.19 | 0.03440 | 0.05160 |
|  |  | RD*APOE | 119.83 | 47.84 | 2.51 | 0.01610 | 0.04830 |
| R^2^_adj_ = 0.3905, F_3,43_ = 10.83, *p* = 0.00002004 | | | | | | | |
| APOE e4 simple slope = 92.67, S.E. = 31.40, *p* = 0.01 | | | | | | | |
| CPL | Left SLF | Intercept | -41.76 | 17.49 | -2.39 | 0.02140 |  |
|  |  | AxD | 44.87 | 15.93 | 2.82 | 0.00731 | 0.00731 |
|  |  | APOE | -104.73 | 34.97 | -2.99 | 0.00455 | 0.00683 |
|  |  | AxD*APOE | 104.03 | 31.87 | 3.26 | 0.00216 | 0.00648 |
| R^2^_adj_ = 0.4582, F_3,43_ = 13.97, *p* = 0.000001699 | | | | | | | |
| APOE e4 simple slope = 96.88, S.E. = 24.47, *p* < 0.01 | | | | | | | |
| EE Score | Left UF | Intercept | -12.26 | 9.27 | -1.32 | 0.19320 |  |
|  |  | AxD | 12.04 | 8.14 | 1.48 | 0.14660 | 0.14660 |
|  |  | APOE | -42.87 | 18.55 | -2.31 | 0.02580 | 0.03870 |
|  |  | AxD*APOE | 40.99 | 16.29 | 2.52 | 0.01580 | 0.03870 |
| R^2^_adj_ = 0.379, F_3,42_ = 10.16, *p* = 0.00003728 | | | | | | | |
| APOE e4 simple slope = 32.54, S.E. = 12.55, *p* = 0.01 | | | | | | | |
| CPL | Left UF | Intercept | -27.72 | 22.90 | -1.21 | 0.23297 |  |
|  |  | AxD | 30.68 | 20.11 | 1.53 | 0.13460 | 0.13460 |
|  |  | APOE | -123.68 | 45.80 | -2.70 | 0.00995 | 0.01493 |
|  |  | AxD*APOE | 116.73 | 40.22 | 2.90 | 0.00588 | 0.01493 |
| R^2^_adj_ = 0.3899, F_3,42_ = 10.59, *p* = 0.00002596 | | | | | | | |
| APOE e4 simple slope = 89.05, S.E. = 30.98, *p* = 0.01 | | | | | | | |

Unadjusted and adjusted p-values reported. Values were adjusted for multiple comparisons using the Holm correction method and were considered statistically significant at *p* < 0.05. S.E., standard error; EE score, endpoint error score; CPL, corrective path length; APOE, apolipoprotein E; FA, fractional anisotropy; MD, mean diffusivity; RD, radial diffusivity; AxD, axial diffusivity; CGC, cingulum cingulate; IFOF, inferior fronto-occipital fasciculus; ILF, inferior longitudinal fasciculus; SLF, superior longitudinal fasciculus; UF, uncinate fasciculus.
